# Adjuvant Capecitabine Following Concurrent Chemoradiotherapy in Locoregionally Advanced Nasopharyngeal Carcinoma: A Randomized Clinical Trial

**DOI:** 10.1001/jamaoncol.2022.4656

**Published:** 2022-10-13

**Authors:** Jingjing Miao, Lin Wang, Sze Huey Tan, Jin-gao Li, Junlin Yi, Enya H.W. Ong, Laura L.Y. Tan, Ye Zhang, Xiaochang Gong, Qiuyan Chen, Yan-qun Xiang, Ming-yuan Chen, Ying Guo, Xing Lv, Wei-xiong Xia, Linquan Tang, Xiaowu Deng, Xiang Guo, Fei Han, Hai-qiang Mai, Melvin L. K. Chua, Chong Zhao

**Affiliations:** 1 Department of Nasopharyngeal Carcinoma, Sun Yat-sen University Cancer Center, State Key Laboratory of Oncology in South China, Collaborative Innovation Center for Cancer Medicine, Guangdong Key Laboratory of Nasopharyngeal Carcinoma Diagnosis and Therapy, Guangzhou, Guangdong, PR China; 2 Division of Clinical Trials & Epidemiological Sciences, National Cancer Centre Singapore, Singapore; 3 Oncology Academic Programme, Duke-NUS Medical School, Singapore; 4 Department of Radiation Oncology, Jiangxi Cancer Hospital of Nanchang University, Nanchang, China; 5 National Health Commission Key Laboratory of Personalized Diagnosis and Treatment of Nasopharyngeal Carcinoma, Jiangxi Cancer Hospital of Nanchang University, Nanchang, China; 6 Department of Radiation Oncology, National Cancer Center/National Clinical Research Center for Cancer/Cancer Hospital, Chinese Academy of Medical Sciences and Peking Union Medical College, Beijing, 100021, PR China; 7 Division of Medical Sciences, National Cancer Centre Singapore, Singapore; 8 Department of Head and Neck and Thoracic Cancers, Division of Radiation Oncology, National Cancer Centre Singapore, Singapore; 9 Ministry of Health Holdings, Singapore; 10 Department of Clinical Trials Center, Sun Yat-sen University Cancer Center, State Key Laboratory of Oncology in South China, Collaborative Innovation Center for Cancer Medicine, Guangdong Key Laboratory of Nasopharyngeal Carcinoma Diagnosis and Therapy, Guangzhou, Guangdong, PR China; 11 Department of Radiation Oncology, Sun Yat-sen University Cancer Center, State Key Laboratory of Oncology in South China, Collaborative Innovation Center for Cancer Medicine, Guangdong Key Laboratory of Nasopharyngeal Carcinoma Diagnosis and Therapy, Guangzhou, Guangdong, PR China

## Abstract

**Importance:**

Induction or adjuvant chemotherapy with concurrent chemoradiotherapy (CCRT) are first-line treatment options for treatment of locoregionally advanced nasopharyngeal carcinoma (LA-NPC). Adjuvant platinum regimens are, however, poorly tolerated, highlighting the unmet need for an efficacious, tolerable adjuvant regimen.

**Objective:**

To investigate the efficacy and safety of adjuvant capecitabine with CCRT for the treatment of patients with LA-NPC.

**Design, Setting, and Participants:**

This open-label randomized clinical trial recruited patients from March 31, 2014, to July 27, 2018, at 3 institutions in China, with at least 3 years of follow-up. The data collection cutoff date was February 9, 2022. Eligibility included stage III-IVb nasopharyngeal carcinoma and at least 1 of the following: T3-4N2 or T1-4N3; plasma Epstein-Barr virus DNA titer higher than 20 000 copies/mL; primary gross tumor volume larger than 30.0 cm^3^; fluorodeoxyglucose F 18 positron emission tomography/computed tomography maximum standard uptake value of the primary gross tumor volume larger than 10.0; or multiple nodal metastases and any larger than 4.0 cm.

**Interventions:**

Patients were randomly assigned 1:1 to receive either capecitabine (1000 mg/m^2^ twice daily for 14 days every 3 weeks for 8 cycles) or observation following CCRT (100 mg/m^2^ cisplatin every 3 weeks for 2 to 3 cycles, depending on duration of radiotherapy).

**Main Outcomes and Measures:**

Failure-free survival in the intention-to-treat cohort was assessed using Kaplan-Meier survival curves compared with the log-rank test. Unstratified Cox proportional hazards regression models were used to estimate hazard ratios, with corresponding 95% CIs based on the Wald test.

**Results:**

There were 180 patients enrolled (median [IQR] age, 47 [40-55] years; 143 [79.4%] men). Among 90 patients in the capecitabine group, 76 (84.4%) had at least 2 high-risk factors; among 90 patients in the control group, 80 (88.9%) had at least 2 high-risk factors. All patients completed CCRT, except 1 patient in the capecitabine group who received 1 cycle of cisplatin. Of the 90 patients in the capecitabine group, 85 (94.4%) received capecitabine, with 71 (78.9%) completing 8 cycles. With a median (IQR) follow-up of 58.0 (49.5-80.1) months, 18 events were recorded in the capecitabine group vs 31 events in the control group. Failure-free survival was improved with adjuvant capecitabine (3 years, 83.3% vs 72.2%; 5 years, 78.5% vs 65.9%; hazard ratio, 0.53 [95% CI, 0.30-0.94]; *P* = .03). The incidence of grade 3 treatment-related adverse events (TRAEs) was higher in the capecitabine group than in the control group (54 of 90 patients [60.0%] vs 46 of 90 patients [51.1%]). Treatment-related adverse events included xerostomia (17 [18.9%] vs 9 [10.0%] patients), mucositis (21 [23.3%] vs 15 [16.7%] patients), and anorexia (8 [8.9%] vs 4 [4.4%] patients). The incidence of grade 3 delayed treatment-related adverse events was comparable in both groups (9 of 83 [10.8%] vs 7 of 81 [8.6%] patients).

**Conclusions and Relevance:**

In this randomized clinical trial, adjuvant capecitabine at the full dose following CCRT was well tolerated and improved failure-free survival among patients with LA-NPC and high-risk factors. Further investigations assessing optimal dose and duration are warranted.

**Trial Registration:**

ClinicalTrials.gov Identifier: NCT02143388

## Key Points

**Question:**

Is the addition of adjuvant capecitabine safe, and does it improve failure-free survival (FFS) among patients with locoregionally advanced nasopharyngeal carcinoma (LA-NPC) who receive concurrent chemoradiotherapy?

**Findings:**

This multicenter, open-label randomized clinical trial of 180 patients with LA-NPC met its primary end point of FFS benefit in favor of the addition of adjuvant capecitabine to concurrent chemoradiotherapy for treatment of patients with LA-NPC. The capecitabine compliance rate was 79%; the incidence of grade 3 or 4 treatment-related adverse events was 61% (55 of 90) in the capecitabine group and 52% (47 of 90) in the group receiving concurrent chemoradiotherapy alone.

**Meaning:**

These findings suggest that treatment with adjuvant capecitabine confers an FFS benefit and is well tolerated in patients with LA-NPC, although future trials should determine optimal dosing and duration of adjuvant capecitabine.

## Teaser

This multicenter, open-label randomized clinical trial conducted in China compares the efficacy and safety of adjuvant capecitabine with concurrent chemoradiotherapy vs concurrent chemoradiotherapy alone for treatment of patients with locoregionally advanced nasopharyngeal carcinoma.

## Introduction

Nasopharyngeal carcinoma (NPC) is a unique head and neck cancer endemic in East and Southeast Asia,^1,2^ where it is invariably associated with Epstein-Barr virus (EBV).^3,4,5,6^ Most patients with NPC present with locoregionally advanced disease (LA-NPC), harboring a proclivity for metastatic recurrences to the bones, liver, and lungs.^7^ Induction chemotherapy or adjuvant chemotherapy in addition to concurrent chemoradiotherapy (CCRT) is the recommended standard of care in high-risk LA-NPC, defined as T3-4N+ or N2-3 in previous randomized clinical trials.^8,9,10,11^ The updated network meta-analysis of chemotherapy in NPC report^12^ supports the efficacy of either systemic intensification strategy for superior survival in LA-NPC.

The role of adjuvant chemotherapy in LA-NPC, using regimens such as cisplatin with fluorouracil^9,13,14,15,16^ or gemcitabine,^17^ gemcitabine and paclitaxel,^18^ and capecitabine at metronomic dosing^19^ has been investigated. The use of adjuvant fluorouracil and gemcitabine in combination with cisplatin yielded no difference in failure-free survival (FFS) and overall survival (OS), due in part to its inferior tolerability compared with induction chemotherapy, with only 50% to 75% of patients able to receive the full treatment dose.^9,15,16,17^ Chen et al^9^ did not demonstrate a survival benefit using adjuvant fluorouracil with cisplatin after follow-up of approximately 5 years^16^ despite enriching for a high-risk subset of patients with LA-NPC; of note, only 62.9% of participants tolerated 3 cycles of adjuvant fluorouracil with cisplatin. Although superiority of adjuvant fluorouracil with cisplatin was not demonstrated, it cannot be concluded based on that trial that CCRT alone was noninferior to CCRT and adjuvant fluorouracil with cisplatin.

Capecitabine has proven single-agent activity in recurrent metastatic NPC and is efficacious when combined with cisplatin for induction chemotherapy in patients with LA-NPC.^20,21,22,23,24,25^ In addition, a recent randomized clinical trial of adjuvant capecitabine given at metronomic dosing for 1 year conferred FFS and OS benefits among patients with high-risk LA-NPC.^19^ Here, we report the efficacy and safety results after a median follow-up of 58.0 months in a randomized clinical trial of adjuvant capecitabine given at the full dose for 6 months.

## Methods

### Study Design and Participants

The trial was designed by investigators from 4 institutions (Trial Protocol in Supplement 1), and patients were recruited from 3 centers in China (eTable 1 in Supplement 2). Eligibility criteria were (1) histologically confirmed, newly diagnosed, nonkeratinizing NPC; (2) age 18 to 70 years; (3) Karnofsky performance status of at least 80 points; (4) TNM stage III-IVb based on American Joint Committee on Cancer/Union for International Cancer Control (AJCC/UICC) 7th edition stage classification system^26^; (5) no prior radiotherapy, chemotherapy, or surgery (except for diagnostic investigations) for the tumor; (6) adequate organ function; and (7) presence of 1 or more of the following unfavorable prognostic factors: T3-4N2 or T1-4N3^27^; plasma EBV DNA titer higher than 20 000 copies/mL^28^; primary gross tumor volume (GTV) greater than 30.0 cm^3,29^; a maximum standard uptake value of primary GTV higher than 10.0 on fluorodeoxyglucose F 18 positron emission tomography/computed tomography (^18^F-FDG PET/CT)^30^; or multiple nodal metastases and at least 1 larger than 4.0 cm.^31^ Exclusion criteria included (1) prior malignant tumors within 5 years of NPC diagnosis; (2) concurrent pregnancy; (3) concurrent immunotherapy or hormone therapy for other diseases; and (4) severe comorbidities. The protocol was reviewed and approved by the Ethics Committee of Sun Yat-sen University Cancer Center (SYSUCC) and by ethics committees and institutional review boards of the other participating hospitals on March 19, 2014. The study was registered on ClinicalTrials.gov May 7, 2014,^32^ and was performed in accordance with the Declaration of Helsinki and Good Clinical Practice guidelines. The first patient was recruited on March 31, 2014, and the second patient was recruited on July 23, 2014. Trial accrual was completed on July 27, 2018. Data analyses were performed based on a data collection cutoff date of February 9, 2022. All patients provided written informed consent. No one received compensation or was offered any incentive for participating in this study. This study followed the Consolidated Standards of Reporting Trials (CONSORT) reporting guideline.

### Randomization and Masking

Patients were randomly assigned to receive in a 1:1 ratio either capecitabine (capecitabine group) or observation (control group) following CCRT. Randomization was performed at the Clinical Trials Center of SYSUCC on the day of study recruitment prior to CCRT. A computer-generated sequence was used to obtain the randomization list without stratification. After obtaining informed consent, the investigators at each institution contacted the study coordinators at the Clinical Trials Center and received treatment assignment information. The statistician (Y.G.) and study coordinators were uninvolved in the treatment of patients and data monitoring.

### Procedures

All patients underwent CCRT and received intravenous cisplatin at a dosage of 100 mg/m^2^ every 3 weeks for 2 to 3 cycles depending on the duration of radiotherapy. Patients randomized to the capecitabine group then received 8 cycles of oral capecitabine at a dosage of 1000 mg/m^2^ twice daily for 14 days every 21 days,^21^ commencing 4 weeks after CCRT. Chemotherapy dose modifications were based on the nadir blood counts and acute toxic effects of the preceding cycle.

All patients received intensity-modulated radiotherapy (IMRT), based on the principles of ICRU (International Commission on Radiation Units and Measurements) reports 50 and 62. Details of the IMRT technique and delineation of target volumes, including primary GTV, GTV of all involved lymph nodes, high- and low-risk clinical target volumes, and the corresponding planning target volumes, were as previously described.^33^ Prescribed doses were 68 to 72 Gy, 60 to 68 Gy, 60 to 64 Gy, and 54 to 58 Gy in 30 to 32 fractions to the primary planning target volume, lymph node planning target volume, high-risk planning target volume, and low-risk planning target volume, respectively. Plan review was performed centrally by study investigators (J.-G.L., J.Y., H.-Q.M., and C.Z.).

Patients underwent the following evaluations within 2 weeks before randomization: physical examination and nasopharyngoscopy, Karnofsky performance status evaluation, hematologic and biochemical analyses, EBV DNA titer, magnetic resonance imaging or CT of the head and neck, and ^18^F-FDG PET/CT. If ^18^F-FDG PET/CT was unavailable, CT of the chest and abdomen and skeletal scintigraphy were performed. Plasma EBV DNA assays were performed centrally at SYSUCC for standardization.

Trial participants were reviewed at the following intervals: weekly during IMRT, prior to each cycle of capecitabine, every 3 months for the first 3 years, and every 6 months thereafter. All end points were assessed and validated by the physician in charge. The surveillance protocol included hematologic and biochemical analyses, contrast-enhanced magnetic resonance imaging or CT of the head and neck region, and CT of the thorax and abdomen 3 and 6 months after CCRT and annually thereafter. A plasma EBV DNA assay was performed during the last week of CCRT, and nasopharyngoscopy was performed 1 month after CCRT to evaluate tumor response. When the presence of recurrence was indeterminate, ^18^F-FDG PET/CT was performed. When feasible, fine-needle aspiration or biopsy was performed for patients with suspected recurrence. Salvage treatments for recurrent disease were at the discretion of the physician in charge.

### Outcomes

The primary end point was FFS, defined as time from the date of randomization to a documented relapse or any death. Patients unavailable for follow-up or alive without disease relapse were censored at the date of last follow-up. Secondary end points included OS, defined as time from the date of randomization to any death, with patients unavailable for follow-up censored at the date of last follow-up; distant metastasis–free survival (DMFS), defined as time from date of randomization to documented distant metastasis or any death; and locoregional relapse–free survival (LRFS), defined as time from the date of randomization to documented locoregional relapse or any death. Patients with distant metastasis as a first event were censored for LRFS at the date of distant metastasis and vice versa; if distant metastasis and locoregional relapse occurred concurrently, patients were considered as having an event for both DMFS and LRFS. Patients unavailable for follow-up or alive without distant metastasis or locoregional relapse were censored at the date of last follow-up.

Treatment compliance and treatment-related adverse events (TRAEs) during and after treatment were also recorded. For the capecitabine group, TRAEs were assessed at baseline and prior to each chemotherapy cycle until capecitabine completion or at the time of treatment discontinuation. For the control group, TRAEs were assessed at baseline, prior to each concurrent cisplatin cycle and 3 and 6 months after CCRT. Grading of TRAEs was performed using the Common Toxicity Criteria for Adverse Events, version 4.0, and the Radiation Therapy Oncology Group radiation morbidity scoring criteria. Delayed TRAEs were assessed using the latter.^34^

### Statistical Analysis

The sample size for this study was calculated using the PS: Power and Sample Size Calculation, version 3.1.2, software. A 2-sided log-rank test with 54 events in both groups provided at least 80.0% power to detect a hazard ratio (HR) of 0.46 when the 3-year FFS rate in the control group was 70.0% (estimated based on historical data^35,36,37^) at a significance level of 5.0%. This HR would correspond to a 15.0% improvement in 3-year FFS with the addition of adjuvant capecitabine to CCRT.^38^ Considering a 4-year recruitment period and 3 years of follow-up, 164 patients (82 per group) were needed, assuming the distribution of survival times for both groups would follow an exponential distribution. This yielded a total cohort size of 180 patients (90 per group), accounting for a dropout rate of 10.0%.

Primary efficacy analysis was performed based on the intention-to-treat (ITT) principle. Safety data were summarized for all patients who started the assigned treatment. Survival curves were derived using the Kaplan-Meier method and compared using the log-rank test. Unstratified Cox proportional hazards regression models were used to estimate HR. Corresponding 95% CIs were based on the Wald test. The proportional hazards assumption was tested by including time-dependent covariates in the Cox models within the PROC PHREG module (test for interaction), and by assessing whether the Schoenfeld residuals were independent of time using simple linear regression and a trend test. Graphical methods were used to check the proportional hazards assumption using smoothed plots of scaled Schoenfeld residuals against time. Linear and log time scales were used for the assessment of proportional hazards.^39,40^ Survival rates at 3 and 5 years were reported with corresponding 95% CIs calculated using log transformation of survival probabilities. Median follow-up time was estimated using the reverse Kaplan-Meier method.

At the data collection cutoff date of February 9, 2022, the prespecified number of events (54 events) for the primary end point was not reached. The trial steering committee opted to report the results on the following grounds: there was (1) a lower than expected number of events despite enriching for a high-risk study population, (2) there was a substantial decrease in events beyond the third year of follow-up (9 vs 40 events in the first 3 years), and (3) the study period had reached 8 years with a median follow-up of nearly 5 years and a minimum follow-up of 42.5 months for the last patient enrolled.

A 2-sided *P* < .05 was considered statistically significant. Statistical analyses were performed using SAS, version 9.4 (SAS Institute Inc); STATA, version 16 (StataCorp LLC); and R*,* version 4.0.2 (R Foundation for Statistical Computing). Figures were drawn using GraphPad Prism, version 9.0.0 (GraphPad Software).

## Results

### Patient and Treatment Characteristics

**Figure 1.**
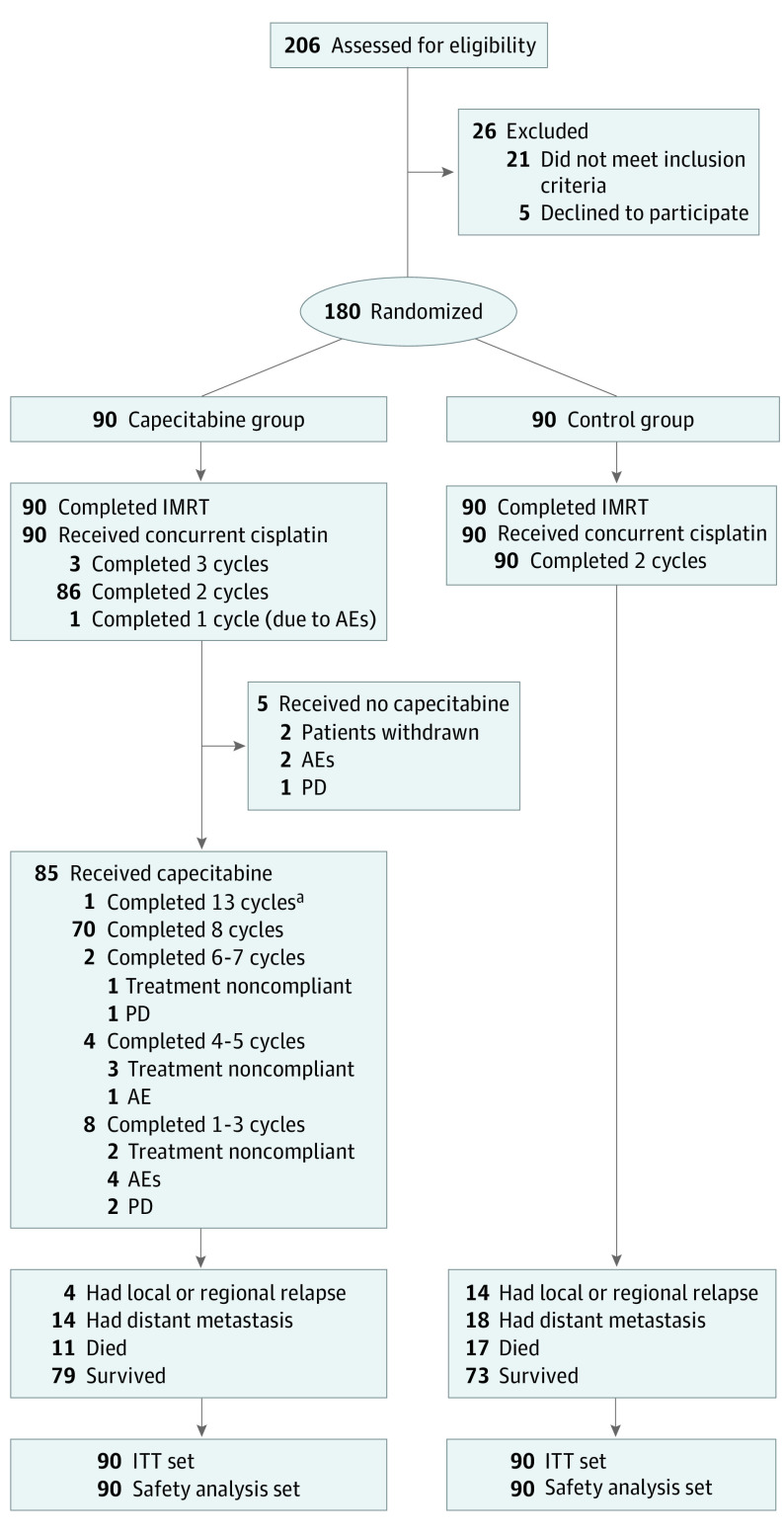
Trial Profile All patients randomly assigned to either adjuvant capecitabine (capecitabine group) or observation (control group) following concurrent chemoradiotherapy were included in the intention-to-treat (ITT) analysis according to their allocated treatments. All patients who received at least 1 dose of chemotherapy were included in the safety analysis. AEs represent adverse events; IMRT, intensity-modulated radiotherapy; PD, progressive disease. ^a^Patient purchased adjuvant capecitabine off protocol from another hospital.

Between March 31, 2014, and July 27, 2018, 206 patients were screened, of whom 180 patients (median [IQR] age, 47 [40-55] years; 143 [79.4%] men; 37 [20.6%] women; and 156 [86.7%] with ≥2 unfavorable risk factors) were enrolled and randomly assigned to the capecitabine group (median [IQR] age, 46 [39-55] years; 73 [81.1%] men and 17 [18.9%] women) or the control group (median [IQR] age, 48 [41-55] years; 70 [77.8%] men and 20 [22.2%] women) (Figure 1). Pretreatment clinical characteristics were comparable between groups (Table 1). Of 90 patients in the capecitabine group, 14 (15.6%) had 1 unfavorable risk factor, 31 (34.4%) had 2 risk factors, and 45 (50.0%) had 3 or more risk factors. Of 90 patients in the control group, 10 (11.1%) had 1 unfavorable risk factor, 42 (46.7%) had 2 risk factors, and 38 (42.2%) had 3 or more risk factors (Table 1; eTable 2 and eFigure 1 in Supplement 2).

**Table 1.** Patient Pretreatment Clinical and Demographic Characteristics, and Treatment Summary and Response Rates Before Starting Capecitabine

	Group, No. (%)	
	Capecitabine (n = 90)	Control (n = 90)
**Pretreatment characteristic** ^a^		
Age, median (IQR), y	46 (39-55)	48 (41-55)
Sex		
Female	17 (18.9)	20 (22.2)
Male	73 (81.1)	70 (77.8)
KPS score, points		
80	2 (2.2)	3 (3.3)
≥90	88 (97.8)	87 (96.7)
WHO category		
II	10 (11.1)	5 (5.6)
III	80 (88.9)	85 (94.4)
T stage^b^		
T1	3 (3.3)	2 (2.2)
T2	7 (7.8)	5 (5.6)
T3	53 (58.9)	56 (62.2)
T4	27 (30.0)	27 (30.0)
N stage^b^		
N0	3 (3.3)	2 (2.2)
N1	27 (30.0)	26 (28.9)
N2	38 (42.2)	45 (50.0)
N3	22 (24.4)	17 (18.9)
TNM stage^b^		
III	45 (50.0)	49 (54.4)
IVa	23 (25.6)	24 (26.7)
IVb	22 (24.4)	17 (18.9)
No. of high-risk factors		
1	14 (15.6)	10 (11.1)
2	31 (34.4)	42 (46.7)
≥3	45 (50.0)	38 (42.2)
TNM stage classification^b^		
T3-4N2M0	35 (38.9)	42 (46.7)
T1-4N3M0	22 (24.4)	17 (18.9)
All others	33 (36.7)	31 (34.4)
SUV_max_		
≤10.0	13 (14.4)	16 (17.8)
>10.0	67 (74.4)	66 (73.3)
Not performed	10 (11.1)	8 (8.9)
Primary GTV, cm^3^		
≤30.0	20 (22.2)	21 (23.3)
>30.0	70 (77.8)	69 (76.7)
Max LN diameter, cm		
≤4.0	83 (92.2)	81 (90.0)
>4.0	7 (7.8)	9 (10.0)
EBV DNA titer, copies/mL		
≤20 000	74 (82.2)	73 (81.1)
>20 000	15 (16.7)	16 (17.8)
Not performed	1 (1.1)	1 (1.1)
**Treatment**		
Receipt of radiotherapy	90 (100)	90 (100)
With radiotherapy delay	36 (40.0)	29 (32.2)
Radiotherapy delay, d	1 (1-4)	1 (1-2)
No. of cisplatin cycles completed		
1	1 (1.1)^c^	0
2	86 (95.6)	90 (100)
3	3 (3.3)	0
Cumulative cisplatin dose intensity, median (IQR), mg/m^2^	200.0 (200.0-200.0)	200.0 (200.0-200.0)
<200.0	10 (11.1)	4 (4.4)
≥200.0	80 (88.9)	86 (95.6)
Patients with cisplatin administration delay	30 (33.3)^c^	33 (36.7)
Time until start capecitabine, d	28 (27-31)	NA
**Response**		
Objective response rate after CCRT^d^		
Complete response	78 (86.7)	79 (87.8)
Partial response	12 (13.3)	10 (11.1)
Could not be assessed	0	1 (1.1)^e^
EBV DNA titer after CCRT, copies/mL		
0	79 (87.7)	76 (84.4)
>0	7 (7.8)	9 (10.0)
Not performed	4 (4.4)	5 (5.6)

Abbreviations: CCRT, concurrent chemoradiotherapy; EBV, Epstein-Barr virus; GTV, gross tumor volume; KPS, Karnofsky performance status; Max LN, maximum lymph node; NA, not applicable; SUV_max_, maximum standard uptake value; WHO, World Health Organization.

^a^ Distribution of eligibility criteria well balanced between groups (details in eFigure 1 and eTable 2 in Supplement 2).

^b^ American Joint Committee on Cancer/Union for International Cancer Control, 7th edition.

^c^ One patient had grade 2 hepatotoxicity after receiving 1 cycle of cisplatin.

^d^ Objective response rate by nasopharyngoscopy at 1 month after CCRT, prior to commencement of capecitabine.

^e^ One patient committed suicide 1 week after CCRT.

All patients completed the prescribed course of IMRT, with comparable dosimetric outcomes (eTable 3 in Supplement 2). In the capecitabine group, 89 of 90 patients (98.9%) completed at least 2 cycles of concurrent cisplatin (1 patient received only 1 cycle due to grade 2 hepatotoxicity). All patients in the control group completed 2 cycles of concurrent cisplatin (Figure 1). The median (IQR) cumulative dose intensity for cisplatin in both groups was 200.0 (200.0-200.0) mg/m^2^ (Table 1).

Of 90 patients in the capecitabine group, 85 (94.4%) received capecitabine, with 71 (78.9%) completing all 8 cycles; 5 patients did not initiate treatment, 8 patients completed 1 to 3 cycles, 4 patients completed 4 to 5 cycles, and 2 patients completed 6 to 7 cycles. The median (IQR) relative dose intensity of capecitabine was 100% (84.7%-100%) (eTable 4 in Supplement 2). The capecitabine dose was reduced for 19 of 90 patients (21.1%): 11 because of hand-foot syndrome, 4 because of gastrointestinal adverse events, 2 because of recurrent myelosuppression, 1 because of fatigue, and 1 because of treatment noncompliance.

### Outcomes

At 1 month after CCRT, endoscopic examination indicated that for 90 patients in the capecitabine group, 78 (86.7%) showed complete response and 12 (13.3%) showed partial response; and for 90 patients in the control group, 79 (87.8%) showed complete response and 10 (11.1%) showed partial response (Table 1). The median (IQR) follow-up duration was 58.0 (49.5-80.1) months. The last patient enrolled in the trial was followed up for 42.5 months. We recorded 49 disease relapse or death events: 18 in the capecitabine group and 31 in the control group. Details regarding the patterns of relapse and salvage therapies are shown in eTables 5 and 6 in Supplement 2. Of note, 7 patients in the capecitabine group and 5 patients in the control group received anti–programmed cell death 1 antibodies.

**Figure 2.**
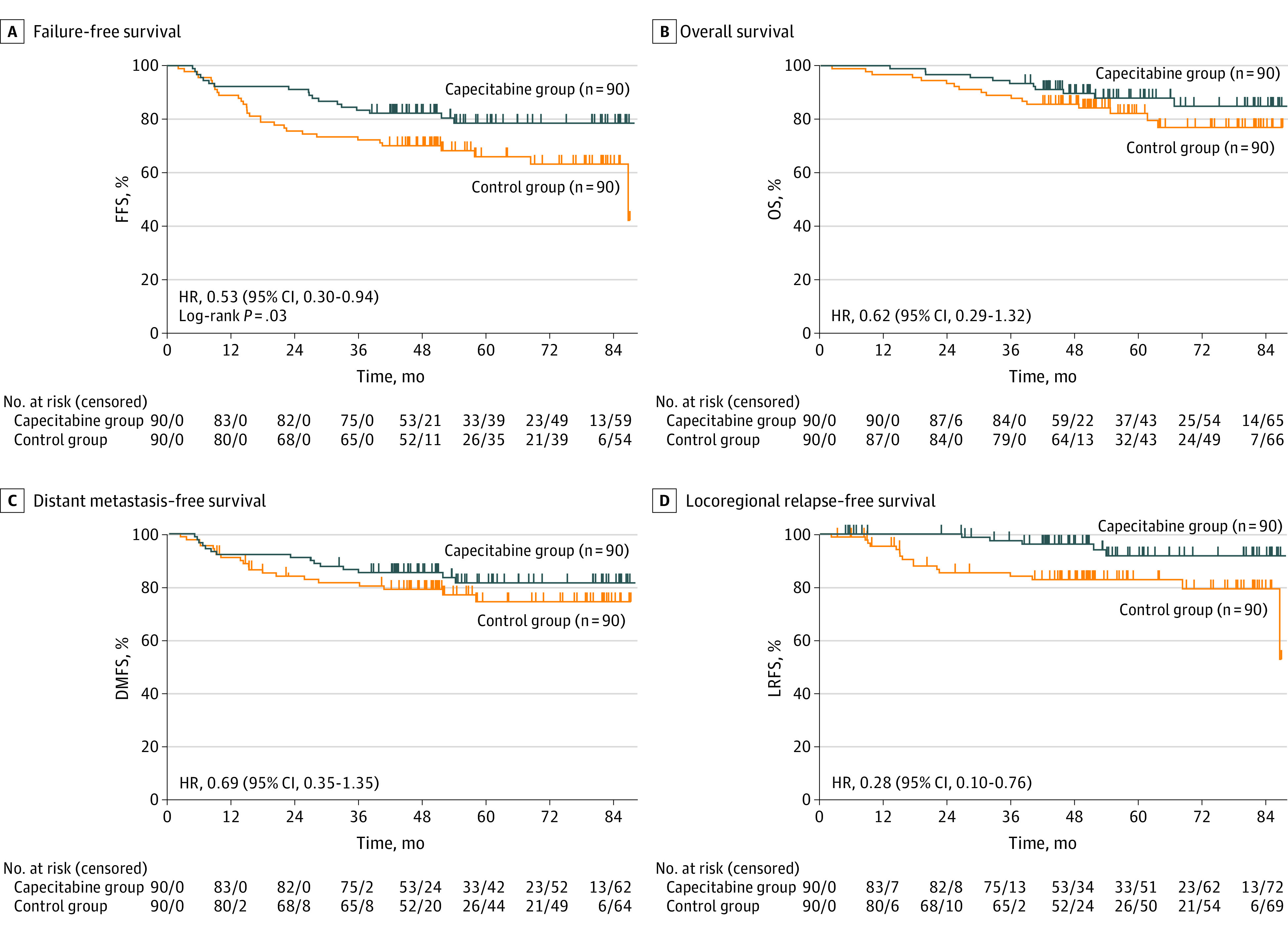
Survival Curves in the Intention-to-Treat Set DMFS represents distant metastasis–free survival; FFS, failure-free survival; HR, hazard ratio; LRFS, locoregional relapse–free survival; and OS, overall survival.

For the primary end point in the ITT set, treatment with CCRT and capecitabine resulted in FFS superior to treatment with CCRT only (3 years, 83.3% vs 72.2%; 5 years, 78.5% vs 65.9%; HR, 0.53 [95% CI, 0.30-0.94]; *P* = .03) (Figure 2A; Table 2; eFigure 2 in Supplement 2). The rates for 3-year OS were 93.3% for the capecitabine group vs 87.8% for the control group (HR, 0.62; 95% CI, 0.29-1.32); rates for 3-year DMFS were 85.5% for the capecitabine group vs 80.4% for the control group (HR, 0.69; 95% CI, 0.35-1.35); and rates for 3-year LRFS were 97.5% for the capecitabine group vs 84.1% for the control group (HR, 0.28; 95% CI, 0.10-0.76) (Figure 2B-D; Table 2; eFigure 2 in Supplement 2).

**Table 2.** Survival Analyses for the Intention-to-Treat Set

Survival	Events, No. (%)^a^		Survival at 3 years, % (95% CI)		Survival at 5 years, % (95% CI)	
	Capecitabine (n = 90)	Control (n = 90)	Capecitabine (n = 90)	Control (n = 90)	Capecitabine (n = 90)	Control (n = 90)
Failure-free	18 (20.0)	31 (34.4)	83.3 (73.9-89.6)	72.2 (61.7-80.3)	78.5 (67.7-86.0)	65.9 (54.3-75.2)
Overall	11 (12.2)	17 (18.9)	93.3 (85.8-96.9)	87.8 (79.0-93.0)	87.8 (78.0-93.3)	82.1 (71.7-89.0)
Distant metastasis–free	15 (16.7)	20 (22.2)	85.5 (76.3-91.3)	80.4 (70.3-87.3)	81.6 (70.9-88.7)	74.5 (62.5-83.2)
Locoregional relapse–free	5 (5.6)	16 (17.8)	97.5 (90.4-99.4)	84.1 (74.2-90.5)	91.8 (80.8-96.6)	82.9 (72.7-89.5)

^a^ Events include the survival measure plus any death.

Treatment effects by high-risk factors for FFS in the ITT set are shown in eFigure 3 in Supplement 2. No interaction between the high risk factors and treatment was observed, although the addition of adjuvant capecitabine favored the subgroup of patients having risk factors with a maximum standard uptake value higher than 10.0, a primary GTV larger than 30.0 cm^3^, and an EBV DNA titer higher than 20 000 copies/mL. An exploratory analysis for FFS performed for patients with or without a detectable EBV DNA titer after CCRT suggested an inferior FFS for the former, although statistical significance was not reached (eFigure 4 in Supplement 2).

### TRAEs

Incidences of grade 3 TRAEs during treatment (up to 7 months after CCRT) were higher in the capecitabine group (54 of 90 patients [60.0%]) than for the control group (46 of 90 patients [51.1%]) (Table 3), whereas the incidence of grade 4 TRAEs was 1 patient (1.1%) in each group. Grade 3 TRAEs were mainly xerostomia (17 patients [18.9%] vs 9 patients [10.0%]), mucositis (21 patients [23.3%] vs 15 patients [16.7%]), and anorexia (8 patients [8.9%] vs 4 patients [4.4%]). Among 85 patients, there were 65 occurrences of grade 1 or 2 hand-foot syndrome (76.5%) associated with capecitabine and 3 occurrences of grade 3 (3.5%).

**Table 3.** Incidence of Treatment-Related Adverse Events During Treatment Duration up to 7 Months After Concurrent Chemoradiotherapy

TRAE	Participants, No. (%)			
	Any grade TRAE	Grade 1 or 2 TRAE	Grade 3 TRAE	Grade 4 TRAE
**Capecitabine group (n = 90)** ^a^				
Any TRAEs	90 (100)	35 (38.9)	54 (60.0)	1 (1.1)
Nonhematologic AEs				
Hand-foot syndrome^b^	68 (80.0)	65 (76.5)	3 (3.5)	0
Xerostomia	90 (100)	73 (81.1)	17 (18.9)	0
Mucositis	87 (96.7)	66 (73.3)	21 (23.3)	0
Radiodermatitis	86 (95.6)	84 (93.3)	2 (2.2)	0
Dysgeusia	84 (93.3)	84 (93.3)	0	0
Dysphagia	3 (3.3)	3 (3.3)	0	0
Anorexia	89 (98.9)	81 (90.0)	8 (8.9)	0
Nausea	87 (96.7)	84 (93.3)	3 (3.3)	0
Vomiting	70 (77.8)	67 (74.4)	3 (3.3)	0
Constipation	44 (48.9)	44 (48.9)	0	0
Diarrhea	9 (10.0)	9 (10.0)	0	0
Fatigue	85 (94.4)	85 (94.4)	0	0
Insomnia	16 (17.8)	16 (17.8)	0	0
Allergy	3 (3.3)	3 (3.3)	0	0
Cardiotoxicity	2 (2.2)	2 (2.2)	0	0
Hematologic AEs				
Leukopenia	88 (97.8)	62 (68.9)	26 (28.9)	0
Neutropenia	70 (77.8)	58 (64.4)	11 (12.2)	1 (1.1)
Anemia	69 (76.7)	64 (71.1)	5 (5.6)	0
Thrombocytopenia	23 (25.6)	23 (25.6)	0	0
Hypoalbuminemia	23 (25.6)	23 (25.6)	0	0
Hepatotoxicity	47 (52.2)	46 (51.1)	1 (1.1)	0
Nephrotoxicity	27 (30.0)	27 (30.0)	0	0
**Control group (n = 90)** ^c^				
Any TRAEs	90 (100)	43 (47.8)	46 (51.1)	1 (1.1)
Nonhematologic AEs				
Hand-foot syndrome	NA	NA	NA	NA
Xerostomia	90 (100)	81 (90.0)	9 (10.0)	0
Mucositis	88 (97.8)	73 (81.1)	15 (16.7)	0
Radiodermatitis	86 (95.6)	85 (94.4)	1 (1.1)	0
Dysgeusia	90 (100)	90 (100)	0	0
Dysphagia	1 (1.1)	1 (1.1)	0	0
Anorexia	90 (100)	86 (95.6)	4 (4.4)	0
Nausea	86 (95.6)	83 (92.2)	3 (3.3)	0
Vomiting	68 (75.6)	66 (73.3)	2 (2.2)	0
Constipation	38 (42.2)	38 (42.2)	0	0
Diarrhea	6 (6.7)	6 (6.7)	0	0
Fatigue	85 (94.4)	85 (94.4)	0	0
Insomnia	15 (16.7)	15 (16.7)	0	0
Allergy	2 (2.2)	2 (2.2)	0	0
Cardiotoxicity	1 (1.1)	1 (1.1)	0	0
Hematologic AEs				
Leukopenia	87 (96.7)	57 (63.3)	30 (33.3)	0
Neutropenia	67 (74.4)	49 (54.4)	17 (18.9)	1 (1.1)
Anemia	63 (70.0)	61 (67.8)	2 (2.2)	0
Thrombocytopenia	27 (30.0)	24 (26.7)	3 (3.3)	0
Hypoalbuminemia	17 (18.9)	17 (18.9)	0	0
Hepatotoxicity	40 (44.4)	40 (44.4)	0	0
Nephrotoxicity	29 (32.2)	28 (31.1)	1 (1.1)	0

Abbreviations: AEs, adverse events; NA, not applicable; TRAEs, treatment-related adverse events.

^a^ For the capecitabine group, TRAEs were assessed at baseline and prior to each chemotherapy cycle until capecitabine completion, or at the time of treatment discontinuation.

^b^ Of 85 patients who received at least 1 cycle of adjuvant capecitabine evaluated for hand-foot syndrome.

^c^ For the control group, TRAEs were assessed at baseline, prior to each concurrent cisplatin cycle, and at 3 and 6 months after concurrent chemoradiotherapy, or at the time of treatment discontinuation.

The incidence of grade 3 or 4 delayed TRAEs was 9 of 83 (10.8%) in the capecitabine group and 7 of 81 (8.6%) in the control group (eTable 7 in Supplement 2). The incidences of delayed TRAEs for any grade were comparable between the groups, apart from a higher incidence in the capecitabine group than in the control group of ototoxicity (58 of 83 [69.9%] vs 47 of 81 [58.0%]) and grade 1 xerostomia (49 of 83 [59.0%] vs 40 of 81 [49.4%]).

## Discussion

This randomized clinical trial addressed the unmet clinical need for a tolerable adjuvant regimen after CCRT by showing the efficacy and safety of single-agent adjuvant capecitabine in 180 patients with LA-NPC. With a median follow-up of 58.0 months, we recorded FFS rates in favor of CCRT with adjuvant capecitabine at 3 years (83.3% vs 72.2%) and 5 years (78.5% vs 65.9%; HR, 0.53 [95% CI, 0.30-0.94]; *P* = .03). Of 90 patients, 71 (78.9%) completed the full course of capecitabine, to our knowledge the highest compliance rate reported thus far among adjuvant chemotherapy trials.^9,13,14,15,16,17,19^ Our results support the use of capecitabine as an effective and tolerable adjuvant regimen for treating patients with LA-NPC.

Nevertheless, we acknowledge the relevance of our trial may be questionable with the recent shift toward induction chemotherapy in LA-NPC.^8,41^ At the time of trial design in March 2014, the role of induction chemotherapy was not yet established. Thus, our goal was to find a well-tolerated and effective regimen to add to a CCRT treatment backbone. Chen et al^19^ found that metronomic capecitabine at a dose of 650 mg/m^2^ twice daily for 1 year conferred a 9.6% FFS benefit despite 316 of 406 patients (77.8%) having received induction chemotherapy. That trial, but not ours, showed improved OS, although the cumulative dose intensities of capecitabine in both trials were comparable. The discordance in results thus suggests a need to further investigate the optimal duration and dosing of adjuvant capecitabine in LA-NPC because there could be antiangiogenic and immune modulation properties with metronomic dosing.^42,43^ Patients’ preferences and likelihood of compliance for a 6-month vs a 1-year course of treatment also need to be considered.

Although OS was not significantly different in the ITT population, the 3-year OS difference was 5.5% in favor of adjuvant capecitabine. The lack of statistical significance for OS is a consequence of the low number of death events even though we had enriched for high-risk factors in our study population (156 of 180 patients [86.7%] had ≥2 risk factors). The low number of deaths in our study could be due to the efficacious salvage therapies used at the time of recurrence, which included anti–programmed cell death 1 antibody treatment and local metastasis–directed therapies (eTable 6 in Supplement 2). A meta-analysis of 19 trials investigating the role of CCRT in LA-NPC suggested that progression-free survival may be a justifiable surrogate for OS in this patient population.^42^ This suggestion is further supported by recent trials for patients with NPC that reported FFS and corresponding OS benefits after 3 to 4 years of follow-up.^44,45,46,47^ An updated meta-analysis will provide insights regarding whether progression-free survival remains a valid surrogate for OS among patients with LA-NPC given the emergence of effective salvage therapies.

### Limitations

This study has several limitations. First, this trial did not meet the expected number of FFS events. The trial steering committee opted to report the study results at this juncture because longer follow-up may not have yielded the required number of events given the acute decrease in event rates beyond the third year of follow-up. Second, the high risk factors included in the eligibility criteria were chosen based on retrospective series.^27,28,29,31^ In addition, EBV DNA assay and ^18^F-FDG PET/CT are not widely available in the community, which may limit the applicability of our results. Third, a placebo control and masking of the physicians and patients to the treatments were not used in our trial. Fourth, most trial participants in both groups received only 2 cycles of 100 mg/m^2^ of cisplatin with IMRT. Thus, there is a question of whether adjuvant capecitabine may only be beneficial for patients with high-risk LA-NPC (by the study criteria) who received this deintensified treatment.^48,49^ To clarify, the predominant use of 2 cycles of cisplatin was due to the IMRT fractionation schema, as treatment was completed within 6 to 6.5 weeks. Several studies have reported that cumulative dosing of 160 to 200 mg/m^2^ of cisplatin with radiotherapy is adequate to yield a survival advantage over radiotherapy alone among patients with LA-NPC.^50,51,52^

## Conclusions

The results of this randomized clinical trial indicated that treatment with adjuvant capecitabine following CCRT was well tolerated and had a high compliance rate. Long-term follow-up of nearly 5 years confirmed an FFS benefit with this treatment regimen. These results suggest that single-agent capecitabine may be considered an adjuvant regimen when treating patients with high-risk LA-NPC.

## Notes

Supplement 1.
Trial Protocol

Supplement 2. **eTable 1.** List of Participating Centers **eTable 2.** Distribution of Patients With Different High-Risk Factors in Different Centers **eTable 3.** Dosimetric Outcomes of Radiotherapy Plans in Both Groups **eTable 4.** Compliance to Adjuvant Capecitabine **eTable 5.** Patterns of Failure **eTable 6.** Salvage Treatments After Relapse **eTable 7.** Incidence of Delayed Treatment-Related Adverse Events **eFigure 1.** Distribution of Patients With Different High-Risk Factors **eFigure 2.** Scaled Schoenfeld Residuals Plots for Different Survival End Points **eFigure 3.** Forest Plot for Failure-Free Survival for the Different Patient Subgroups **eFigure 4.** Failure-Free Survival for EBV DNA Titer End-CCRT

Supplement 3.
Data Sharing Statement
