# Supplementary material for: Adjuvant Capecitabine Following Concurrent Chemoradiotherapy in Locoregionally Advanced Nasopharyngeal Carcinoma: A Randomized Clinical Trial
Source: JAMA Oncol. 2022 Oct 13;8(12):1776–85. doi: 10.1001/jamaoncol.2022.4656 (PMC9562101; doi:10.1001/jamaoncol.2022.4656)
Supplement: Supplement 1. — Trial Protocol [file jamaoncol-e224656-s001.pdf]

Protocol

This trial protocol has been provided by the authors to give readers additional information about their work.

Concurrent Cisplatin Chemoradiation with or without Capecitabine as Adjuvant Chemotherapy in Locoregionally Advanced Nasopharyngeal Carcinoma with High-risk Factors: Multicenter Randomized Controlled Clinical Trial.

Clinicaltrials.gov: NCT02143388

Research Sponsor: Sun Yat-sen University Cancer Center, Guangzhou

Principal Investigator: Prof Chong Zhao

Cooperation institutions:

Country	Institution	Investigator
Jiangxi	Jiangxi Cancer Hospital	Jingao Li
Beijing	Cancer Hospital Chinese Academy of Medical Science	Junlin Yi
Singapore	National Cancer Centre Singapore	Melvin L.K. Chua

Contact: Jingjing Miao, Email: miaojj@sysucc.org.cn

TABLE OF CONTENTS

Title page 1
Table of Contents 2
1 Background 3
2 Study Objectives and Endpoints 3
2.1 Primary objective 3
2.2 Secondary objectives 3
2.3 Definition of endpoints 3
3 Patient Eligibility Criteria 4
3.1 Inclusion criteria 4
3.2 Exclusion criteria 4
4 Study Schema 4
5 Treatment Protocol 4
5.1 Radiotherapy 4
5.2 Concurrent chemotherapy 6
5.3 Adjuvant chemotherapy 6
6 Baseline Assessment and Follow-up 7
6.1 Baseline assessment 7
6.2 Assessment during treatment 8
6.3 Follow-up schedule 8
6.4 Salvage treatment 8
7 Ethical and regulatory requirements 8
7.1 Ethical conduct of the study 8
7.2 Patient data protection 8
7.3 Ethics and regulatory review 8
7.4 Informed consent 8
7.5 Changes to the protocol and informed consent form 9
7.6 Audits and inspections 9
8 Statistical Analysis 9
8.1 Sample size calculation 9
8.2 Statistical analysis 9
9 References 10

LIST OF TABLES

Table 1 Delineation of planning target volumes (PTVs) 5
Table 2 Delineation and dose limits of organs at risk (OARs) 5
Table 3 Patient's body surface area (BSA) and corresponding dose of capecitabine 7

LIST OF FIGURES

Figure 1 Study flow chart 4

LIST OF APPENDICES

Appendix A List of abbreviations and definition of terms 11

1 Background

Nasopharyngeal carcinoma (NPC) is a unique type of head and neck cancer with a particular geographical distribution in East and Southeast Asia.[1] In these regions where NPC is endemic, it is often linked to Epstein-Barr virus (EBV) infection.[2] NPC is both a radio- and chemo-sensitive tumor; hence, radiotherapy with or without chemotherapy is the standard treatment modality for non-disseminated NPC. Around 70% of the patients present with locoregionally-advanced NPC (LA-NPC), usually defined as T3-4N+ or N2-3 disease in clinical trials. LA-NPC has a high propensity for distant metastasis to the bones, lungs and liver, and is thus the group of patients that has been targeted for treatment intensification.[3] The standard treatment modality of these patients was concurrent cisplatin chemoradiation (CCRT) followed by adjuvant cisplatin and fluorouracil (PF) chemotherapy.[4-6]

However, the use of adjuvant chemotherapy in treatment for LA-NPC is often challenged by the poor compliance rates.[6-8] Chen *et al*[9] investigated the addition of adjuvant PF chemotherapy to CCRT in a multicenter phase III randomized controlled trial for T3-4N+, N2-3 LA-NPC. The investigators could not demonstrate superiority with adjuvant PF, it must be cautioned that one could not conclude that CCRT alone was non-inferior to CCRT and PF based on these results.[9] Like previous studies, the compliance to the adjuvant chemotherapy in the trial was only 52-61%, with patients having dose reductions and treatment delays, or omitting adjuvant chemotherapy altogether.[6-8] Therefore, there is an unmet need for a drug that can be administered during adjuvant chemotherapy with better tolerability to maximize the efficacy.

Capecitabine is an orally administered prodrug that is metabolize by the liver to its active form 5-fluorouracil, which inhibits DNA synthesis and cell growth. Capecitabine has been found to have significant activity when used as second-line treatment in NPC.[10-12] Chua *et al*[12] performed a stage II clinical trial for recurrent or metastatic NPC patients pre-treated with platinum-based chemotherapy, and found that capecitabine showed a good effect with low toxicity. Therefore, we design this prospective, multicenter, randomized, controlled trial to investigate the efficacy and toxicity of CCRT plus adjuvant capecitabine in LA-NPC patients. Specifically, we enrich for an unfavorable subgroup of LA-NPC patients with at least one of the following unfavorable prognostic factors: (1) T3-4N2 or T1-4N3; (2) plasma EBV DNA titer of >20,000 copies/mL;[13] (3) primary gross tumor volume (GTVp) of >30.0 cm^3;[14] (4) ^{18}F-Fluorodeoxygenase positron emission tomography computed tomography (^{18}F-FDG-PET-CT) maximum standard uptake value (SUV_{max}) of GTVp of >10.0;[15] and (5) multiple nodal metastases and any >4.0 cm,[16] as the eligibility criteria.

2 Study Objectives and Endpoints

2.1 Primary objective

To investigate if adjuvant capecitabine (capecitabine group) improves failure-free survival (FFS) as compared to observation following CCRT (control group) in patients with LA-NPC.

2.2 Secondary objectives

To investigate if capecitabine group improves overall survival (OS), distant metastasis-free survival (DMFS) and locoregional relapse-free survival (LRRFS) as compared to control group; and to compare treatment compliance rates and occurrence of treatment-related adverse events (TRAEs) during treatment duration and post-treatment in capecitabine and control groups.

2.3 Definition of endpoints

1. FFS: defined as the time from the date of randomization to documented relapse or death from any cause.
2. OS: defined as time from date of randomization to death from any cause where patients lost to follow-up were censored at the date of last follow-up.
3. DMFS: defined as the date of randomization to documented distant metastasis or death from any cause.
4. LRRFS: defined as the date of randomization to documented locoregional relapse or death from any cause.

Patients with distant metastasis as a first event will be censored for LRRFS at date of distant metastasis and vice versa; if distant metastasis and locoregional relapse occurred concurrently, patients will be considered as having an event for both DMFS and LRRFS. Patients lost to follow-up or alive without distant metastasis or locoregional relapse will be

censored at the date of last follow-up.

3 Patient Eligibility Criteria

3.1 Inclusion criteria

1. Patients with histologically confirmed, newly-diagnosed non-keratinizing NPC (WHO II/III);
2. Patients between 18 to 70 years old;
3. Patients with Karnofsky Performance Status (KPS) of ≥80 points;
4. Patients with TNM stage III-IVb disease based to the American Joint Committee on Cancer/International Union Against Cancer (AJCC/UICC) 7th edition;
5. Patients with the presence of at least one of the following unfavorable prognostic factors: T3-4N2 or T1-4N3; plasma EBV DNA titer of >20,000 copies/mL;[13] GTVp of >30.0 cm^3;[14] SUV_{max} of >10.0 by ^{18}F-FDG-PET-CT within the primary tumor;[15] or multiple neck node metastases and any >4.0 cm;[16]
6. Patients with no prior radiotherapy, chemotherapy, or surgery (except for diagnostic investigations) to the tumor;
7. Patients with adequate organ function (white blood cell count of $\geq 4.0\times10^9$/L; absolute neutrophil count of $\geq 1.5\times10^9$/L; hemoglobin of ≥100g/L; platelet count of $\geq 100\times10^9$/L; total bilirubin, aspartate aminotransferase and alanine aminotransferase of ≤1.5X the upper limit of normal [ULN]; and creatinine clearance rate of ≥60 mL/min);
8. Patients who provide written informed consent and are amenable for regular follow-up.

3.2 Exclusion criteria

1. Patients with distant metastases;
2. Patients with prior malignancies within 5 years of NPC diagnosis;
3. Patients with drug or alcohol addiction;
4. Patients lacking capacity for providing informed consent;
5. Patients suffering from active systemic infections;
6. Patients concurrent pregnancy or lactation;
7. Patients undergoing concurrent immunotherapy or hormone therapy for other diseases;
8. Patients suffering from severe comorbidities such as poorly controlled diabetes, hypertension, hepatitis and tuberculosis.

4 Study Schema

This is a prospective, multicenter, randomized, controlled trial. 180 patients with LA-NPC will be recruited and randomly assigned in a 1:1 ratio to the capecitabine group or control group.

The study flow chart is presented in **Figure 1**.

Figure 1 Study flow chart

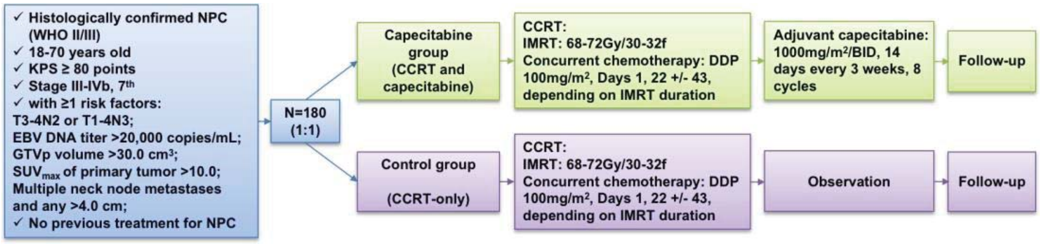

5 Treatment Protocol

5.1 Radiotherapy

5.1.1 Radiotherapy preparation

All patients are immobilized in the supine position using a thermoplastic mask that covered the head, neck, and shoulder. Both non-enhanced computed tomography (CT) (for dose calculation) and contrast enhanced CT (for target delineation) images will be obtained from the vertex to 2.0 cm below the sternoclavicular joint, with 3-mm slices.

5.1.2 Target volume delineation

The target volume delineation includes the GTVp, gross tumor volume of involved lymph nodes (GTVn), high-risk clinical target volume (CTV1) and low-risk clinical target volume (CTV2).

1. The GTVp and GTVn are defined according to the magnetic resonance imaging (MRI) images, as well as clinical and endoscopic findings.
2. The CTV1 is defined as the GTVp plus a 5-10 mm margin (2-3 mm margin posteriorly) to encompass the high-risk sites of microscopic extension and the whole nasopharynx.
3. The CTV2 is defined as the CTV1 plus a 5-10 mm margin (2-3 mm margin posteriorly) to encompass the low-risk sites, and the elective neck area (bilateral levels IIa-b, III, and Va are routinely covered for all N0 patients, whereas ipsilateral levels IV, Vb, or supraclavicular fossae are also included for N1-3 patients).
4. Level Ib will be electively irradiated if: level Ib LNs are involved, level IIa LNs have a diameter ≥3cm or extracapsular extension is present, extensive nodal disease is present on the ipsilateral neck, and the soft or hard palate, oral cavity, or ipsilateral nasal cavity are grossly involved.
5. Planning target volumes (PTVs) are generated automatically after delineation of tumor targets by a uniform expansion ranging from 3 mm (1 mm posteriorly), depending on immobilization and localization uncertainties, as shown in **Table 1**.

Table 1 Delineation of planning Target Volumes (PTVs)

PTVs	Margins
PTVp	GTVp+3mm (1mm posteriorly)
PTVn	GTVn+3mm
PTV1	CTV1+3mm (1mm posteriorly)
PTV2	CTV2+3mm (1mm posteriorly)
Brainstem_PRV	Brainstem+1mm
Spinal cord_PRV	Spinal cord+5mm
Optic nerve_PRV	Optic nerve+1mm
Optic chiasm_PRV	Optic chiasm+1mm

5.1.3 Prescribed dose to tumor target volumes

The prescribed doses are 68-72 Gy/30-32 fractions to PTVp, 60-68 Gy/30-32 fractions to PTVn, 60-64 Gy/30-32 fractions to PTV1, 54-58 Gy/30-32 fractions to PTV2.

The plan will be accepted if:

1. the prescribed dose encompasses at least 95% of the target volume;
2. no greater than 1% of the GTVp receives >95% of the prescribed dose;
3. the maximum dose point is located in the GTVp.

5.1.4 Delineation and dose constraints of organs at risk

All adjacent critical structures, including the brainstem, spinal cord, temporal lobes, lens, optic nerves and chiasm, parotid glands, temporomandibular joints, and mandibles will be carefully outlined.

The maximum dose to these structures is kept within their dose limits, which are listed in **Table 2**.

Table 2 Dose limits of organs at risk (OARs)

OARs	Dose limits
Brainstem_PRV	Dmax ≤56Gy
Spinal cord_PRV	Dmax ≤45Gy
Temporal lobes_PRV	Dmax ≤60Gy
Pituitary	Dmax ≤50Gy
Lens	Dmax ≤5Gy
Optic nerve	Dmax ≤50Gy
Optic chiasm	Dmax ≤50Gy
Temporomandibular joints	Dmax ≤56Gy; D33 ≤45Gy
Mandible	Dmax ≤65Gy; D33 ≤45Gy
Parotids	Dmax ≤60Gy; D33 ≤35Gy

5.1.5 Radiotherapy delay

Radiotherapy will be delayed if patients had ≥grade 3 mucositis or skin reaction and resumed only when these toxicities have recovered ≤grade 2.

5.2 Concurrent chemotherapy

5.2.1 Concurrent chemotherapy regimen

Cisplatin is given at a dose of 100mg/m^2 intravenously, on Days 1, 22 +/- 43 concurrently with IMRT, depending on the duration of radiotherapy.

5.2.2 Dose modification

Dose modifications for hematological and non-hematological toxicities during CCRT will be assessed based on the nadir blood counts and acute toxicities of the preceding cycle.

Cisplatin dose will be reduced by one level (20% [20mg/m^2]) if the patient suffers from the following toxicities:

1. grade 4 neutropenia, febrile neutropenia, neutropenic infection, or related toxicities lasting for more than 7 days;
2. grade 4 thrombocytopenia;
3. creatinine clearance of 40–60mL/min;
4. grade 2 neurotoxicity;
5. grade 3 gastrointestinal toxicity.

5.2.3 Chemotherapy discontinuation

Chemotherapy will be discontinued if the patient suffers from the following toxicities:

1. grade 3 hepatotoxicity (aspartate aminotransferase, alanine aminotransferase, or alkaline phosphatase ≥5.0 times ULN);
2. creatinine clearance of <40mL/min;
3. two episodes of grade 4 diarrhea;
4. grade 3 or higher neurotoxicity.

5.2.4 Toxicity prevention

1. To prevent the nephrotoxic effects of cisplatin, three-day hydration can be administered with cisplatin (on days 0–2); diuretics (mannitol and furosemide) can also be given on the day of cisplatin infusion.
2. To prevent neutropenia, prophylactic granulocyte-colony stimulating factors is permitted except during the first cycle of cisplatin.
3. To prevent chemotherapy-induced nausea and vomiting, an antiemetic regimen of NK-1-receptor antagonist (aprepitant; 125mg on day 1, 80mg on days 2-3), 5-HT3-receptor antagonist (ondansetrone 8mg or granisetron 3mg, intravenously), dexamethasone (10mg intravenously) and metoclopramide (10mg intramuscularly) is recommended.

5.3 Adjuvant chemotherapy

5.3.1 Adjuvant chemotherapy regimen

Oral capecitabine is given 1,000 mg/m^2 twice daily for 14 days every 21 days, commencing at 4 weeks post-CCRT (**Table 3**).

5.3.2 Dose modification

Dose modifications will be based on the nadir blood counts and acute toxicities of the preceding cycle.

1. Grade 1 toxicity: no capecitabine reduction;
2. Grade 2 toxicity:
    a. first occurrence: withdraw oral administration of capecitabine until symptoms have recovered to grade 0-1, and no capecitabine reduction thereafter;
    b. second occurrence: withdraw oral administration of capecitabine until symptoms have recovered to grade 0-1, and capecitabine dose reduction by one level (25%) thereafter;
    c. third occurrence: withdraw oral administration of capecitabine until symptoms have recovered to grade 0-1, and capecitabine dose reduction by two levels (50%) thereafter;
    d. forth occurrence: oral capecitabine will be discontinued.
3. Grade 3 toxicity:
    a. first occurrence: withdraw oral administration of capecitabine until symptoms have recovered to grade 0-1, and capecitabine dose reduction by one level (25%) thereafter;

    b. second occurrence: withdraw oral administration of capecitabine until symptoms have recovered to grade 0-1, and capecitabine dose reduction by two levels (50%) thereafter;
    c. third occurrence: oral capecitabine will be discontinued
4. Grade 4 toxicity:
    a. oral capecitabine will be discontinued.;
    b. if the patient's medical oncologist decides that continuing oral capecitabine is in the patient's best interest, then the drug should be withdrawn until symptoms have recovered to grade 0-1, and capecitabine dose reduction by two levels (50%) thereafter.

Table 3 Patient's body surface area (BSA) and corresponding dose of capecitabine

BSA, m^2	Standard dose, 1,000mg/m^2		
	**Daily dose, mg**	**Morning, no. of pills**	**Night, no. of pills**
<1.375	2,500	3	2
1.375-1.625	3,000	3	3
1.625-1.875	3,500	4	3
1.875-2.225	4,000	4	4
>2.225	4,500	5	4
**BSA, m^2**	**First reduction, 750mg/m^2**		
	**Daily dose, mg**	**Morning, no. of pills**	**Night, no. of pills**
<1.5	2,000	2	2
1.5-1.833	2,500	3	2
1.833-2.16	3,000	3	3
>2.16	3,500	4	3
**BSA, m^2**	**Second reduction, 500mg/m^2**		
	**Daily dose, mg**	**Morning, no. of pills**	**Night, no. of pills**
<1.25	1,000	1	1
1.25-1.75	1,500	2	1
1.75-2.25	2,000	2	2
>2.25	2,500	3	2

5.3.3 Adjuvant chemotherapy discontinuation

Chemotherapy will be discontinued if the patient has the following:

1. grade 3/4 allergy or neurotoxicity;
2. grade 4 hematological toxicity or acute mucositis;
3. unacceptable delays due to prolonged drug toxicity;
4. disease progression;
5. withdrawal from trial voluntarily.

5.3.4 Toxicity prevention

1. To prevent neutropenia, prophylactic granulocyte-colony stimulating factors is permitted;
2. To prevent chemotherapy-induced nausea and vomiting, an antiemetic regimen of NK-1-receptor antagonist (aprepitant; 125mg on day 1, 80mg on days 2-3), 5-HT3-receptor antagonist (ondansetrone 8mg or granisetron 3mg, intravenously), or metoclopramide (10mg intramuscularly) is permitted.

6 Baseline Assessment and Follow-up

6.1 Baseline Assessment

Patients must undergo the following examinations to determine the stage and other baseline parameters within two weeks before randomization:

1. Medical history review, physical examination of the head and neck region, neurological assessment, KPS evaluation.
2. Nasal endoscopy and biopsy for histological confirmation of NPC.
3. Blood routine and biochemistry tests including full blood count, renal panel, liver panel, electrolyte panel, lipid panel, blood glucose and inflammatory factors panel.
4. MRI or CT imaging of the head and neck region.
5. ^{18}F-FDG-PET-CT for distant staging.
6. If ^{18}F-FDG-PET-CT is not performed, conventional workup such as CT of the thorax

and abdomen and emission computed tomography.

7. Plasma EBV DNA assays, which will be centrally performed at the SYSUCC for standardization.

6.2 Assessment During Treatment

Routine physical examination, hematological and biochemical blood tests will be performed weekly during CCRT. Plasma EBV DNA assay will be performed on the last week of CCRT.

For the capecitabine group, TRAEs will be assessed at baseline, and prior to each chemotherapy cycle until capecitabine completion, or at the time of treatment discontinuation. For the control group, TRAEs will be assessed at baseline, prior to each concurrent cisplatin cycle and at 3 and 6 months post-CCRT, or at the time of treatment discontinuation. TRAEs will be graded using the Common Toxicity Criteria for Adverse Events version 4.0 (CTCAE v4.0) for chemotherapy-related toxicities, and the Radiation Therapy Oncology Group (RTOG) radiation morbidity scoring criteria for radiotherapy-related toxicities.

6.3 Follow-up Schedule

All patients will be assessed by physical examination of the head and neck region and nasopharyngoscopy at the following intervals:

1. During years 1-3 post treatment: every three months.
2. During years 4-5 post treatment: every six months.

The nasopharyngoscopy will be performed 1 month post-CCRT to evaluate tumor response.

Hematological and biochemical blood tests, contrast-enhanced CT and/or MRI of the head and neck region performed, and CT of the thorax and abdomen will be performed 3 months post-CCRT, 6 months post-CCRT, and annually thereafter. ^{18}F-FDG-PET-CT can be used to detect distant metastasis at the clinician's discretion.

Delayed TRAEs post-treatment will be assessed using the RTOG radiation morbidity scoring criteria six-monthly during the first year and yearly thereafter.

6.4 Salvage treatment

Biopsy or fine needle aspiration (FNA) should be used to confirmed disease recurrence, if the site is amenable for a biopsy. Salvage treatment for residual disease and/or tumor relapse, if detected, will be tailored on a case-by-case basis and NCCN Guidelines on Head and Neck Cancers and at the discretion of the physician-in-charge.

7 Ethical and Regulatory Requirements

7.1 Ethical conduct of the study

The study will be performed in accordance with ethical principles that have their origin in the Declaration of Helsinki and be consistent with International Conference on Harmonization (ICH)/Good Clinical Practice (GCP), applicable regulatory requirements and the Sun Yat-sen University Cancer Center (SYSUCC) policy on Bioethics and Human Biological Samples.

7.2 Patient data protection

The Informed Consent Form will be a separate document that complies with relevant data protection and privacy legislation. We will not provide any data concerning patients to any insurance company, any employer, their family members, general physician or any other third party, unless required to do so by law.

7.3 Ethics and regulatory review

An Ethics Committee, Independent Ethics Committee or Institutional Review Board (IRB) from SYSUCC, should approve the final study protocol, including the final version of the Informed Consent Form and any other written information and/or materials to be provided to the patients. The investigator will ensure the distribution of these documents to the applicable Ethics Committee, and to the study site staff.

7.4 Informed consent

The Principal Investigator(s) at each institution will:

1. Ensure each patient is given full and adequate oral and written information about the nature, purpose, possible risk and benefit of the study;
2. Ensure each patient is notified that they are free to withdraw from the study at any time;

3. Ensure that each patient is given the opportunity to ask questions and allowed time to consider the information provided;
4. Ensure each patient provides signed and dated informed consent before conducting any procedure specifically for the study;
5. Ensure the original, signed Informed Consent Form(s) is/are stored in the Investigator's Study File;
6. Ensure a copy of the signed Informed Consent Form is given to the patient;
7. Ensure that any incentives for patients who participate in the study as well as any provisions for patients harmed as a consequence of study participation are described in the informed consent form that is approved by an Ethics Committee.

7.5 Changes to the protocol and informed consent form

Study procedures will not be changed without the mutual agreement of all coordinating investigators. If there are any substantial changes to the study protocol, then these changes will be documented in a study protocol amendment and where required in a new version of the study protocol (Revised Clinical Study Protocol). The amendment is to be approved by the relevant Ethics Committee and if applicable, also the national regulatory authority approval, before implementation. Local requirements are to be followed for revised protocols. If a protocol amendment requires a change to an institution's Informed Consent Form, the SYSUCC's Ethics Committee is to approve the revised Informed Consent Form before the revised form is used. If local regulations require, any administrative change will be communicated to or approved by each Ethics Committee.

7.6 Audits and inspections

The Ethics Committee will perform audits or inspections including source data verification. The purpose of an audit or inspection is to systematically and independently examine all study-related activities and documents, to determine whether these activities are conducted, and data are recorded, analyzed, and accurately reported according to the protocol, GCP, guidelines of the ICH, and any applicable regulatory requirements.

8 Statistical Analysis

8.1 Sample size calculation

Sample size of this study is calculated with Power and Sample Size Calculation (PS v3.1.2). A two-sided log-rank test with 54 overall events in both groups will provide at least 80% power to detect a hazard ratio (HR) of 0.46 when the 3-year FFS rate in the control group was 70% (estimated based on historical data[17-19]) at a significance level of 5%. This HR would correspond to a 15.0% improvement in 3-year FFS with the addition of adjuvant capecitabine to CCRT. Considering a 4-year recruitment period and 3 years of follow-up, 164 patients (82 per group) will be needed to observe 54 FFS events, assuming that the distribution of survival times for both groups follow the exponential distribution. A total of 180 patients (90 per group) will be needed accounting for a drop-out rate of 10.0%.

8.2 Statistical analysis

Patient demographics and clinical characteristics that are categorical variables will be summarized as frequencies with percentages, and continuous variables will be summarized as medians with inter-quartile ranges (IQR). Survival curves will be derived using the Kaplan-Meier method and compared using the log-rank test. Unstratified Cox proportional hazards regression models will be used to estimate hazard ratios (HRs). Corresponding 95% confidence intervals (CIs) will be based on the Wald test. The proportional hazards assumption will be tested by including time-dependent covariates in the Cox models within the PROC PHREG module. Survival rates at 3- and 5-year will be reported with corresponding 95% CIs calculated using the log(-log) transformation of survival probabilities. Median follow-up time will be estimated using the reverse Kaplan-Meier method. Two-sided p-value of <0.05 is considered as statistically significant. Statistical analyses will eb performed using the SAS version 9.3 (SAS Institute Inc., Cary, NC) and R version 4.0.2 (http://www.r-project.org). Figures will be drawn using GraphPad Prism version 7.0.0 (GraphPad Software, San Diego, CA).

9 References

1. Cancer incidence in five continents. Volume VIII. *IARC Sci Publ.* 2002;(155):1-781.
2. Chien YC, Chen JY, Liu MY, et al. Serologic markers of Epstein-Barr virus infection and nasopharyngeal carcinoma in Taiwanese men. *New Engl J Med.* 2001; 345:1877–1882.
3. Wei WI, Sham JS. Nasopharyngeal carcinoma. *Lancet.* 2005; 365(9476):2041-54.
4. B Baujat, H Audry, J Bourhis, et al. Chemotherapy in locally advanced nasopharyngeal carcinoma: an individual patient data meta-analysis of eight randomized trials and 1753 patients. *Int J Radit Oncol Biol Phys.* 2006; 64(1):47-56.
5. NCCN Guideline Version 2. 2013, http://www.nccn.org/professionals/physician_gls/ PDF / head-and-neck
6. Al-Sarraf M, LeBlanc M, Giri S, et al. Chemoradiotherapy versus radiotherapy in patients with advanced nasopharyngeal cancer: Phase III randomized intergroup study 0099. *J Clin Oncol.* 1998; 16:1310-1317.
7. Wee J, Tan EH, Tai BC, et al. Randomized trial of radiotherapy versus concurrent chemoradiotherapy followed by adjuvant chemotherapy in patients with American Joint Committee on Cancer/International Union against cancer stage III and IV nasopharyngeal cancer of the endemic variety. *J Clin Oncol.* 2005; 23(27): 6730–6738.
8. Lee AWM, Lau WH, Tung SY, et al. Preliminary Results of a Randomized Study on Therapeutic Gain by Concurrent Chemotherapy for Regionally-Advanced Nasopharyngeal Carcinoma: NPC-9901 Trial by the Hong Kong Nasopharyngeal Cancer Study Group. *J Clin Oncol.* 2005; 23(28):6966-6975.
9. L Chen, CS Hu, XZ Chen, et al. Concurrent chemoradiotherapy plus adjuvant chemotherapy versus concurrent chemoradiotherapy alone in patients with locoregionally advanced nasopharyngeal carcinoma: a phase 3 multicentre randomised controlled trial. *Lancet Oncology.* 2012; 13(2):163-171.
10. Ciuleanu E, Irimie A, Ciuleanu TE, Popita V, Todor N, Ghilezan N. Capecitabine as salvage treatment in relapsed nasopharyngeal carcinoma: a phase II study. *J BUON.* 2008; 13(1):37-42.
11. Chua D, Wei WI, Sham JS, Au GK. Capecitabine monotherapy for recurrent and metastatic nasopharyngeal cancer. *Jpn J Clin Oncol.* 2008; 38(4):244-9.
12. Chua DT, Sham JS, Au GK. A phase II study of capecitabine in patients with recurrent and metastatic nasopharyngeal carcinoma pretreated with platinum-based chemotherapy. *Oral Oncol*. 2003; 39(4):361-6.
13. Hou X, Zhao C, Guo Y, et al. Different Clinical Significance of Pre- and Post-treatment Plasma Epstein–Barr Virus DNA Load in Nasopharyngeal Carcinoma Treated with Radiotherapy. *Clinical Oncology.* 2011; 23:128-133.
14. Zhao C, Han F, Lu L, et al. Pattern of failure and prognostic factors for primary nasopharyngeal carcinoma (npc) treated with intensity modulated radiotherapy (IMRT). *Int J Radiat Oncol Biol Phys.* 2008; 72(1) Supplement:383.
15. Xu AA, Han F, Lu LX, et al. The role of pretreatment FDG standard uptake value in predicting for clinical outcome in locally-advanced nasopharyngeal carcinoma. *Chin J Radiat Oncol.* 2012; 21(3):209-213.
16. JC Lin, WM Liang, JS Jan, et al. Another way to estimate outcome of advanced nasopharyngeal carcinoma—is concurrent chemoradiotherapy adequate? *Int J Radiat Oncol Biol Phys.* 2004; 60(1):156-164.
17. Zhao C, Xiao WW, Han F, et al. Long-term outcome and prognostic factors of patients with nasopharyngeal carcinoma treated with intensity-modulated radiation therapy. *Chin J Radiat Oncol.* 2010; 19(3):191-196.
18. Sun X, Zeng L, Chen C, et al. Comparing treatment outcomes of different chemotherapy sequences during intensity modulated radiotherapy for advanced N-stage nasopharyngeal carcinoma patients. *Radiat Oncol.* 2013; 8:265.
19. Su SF, Han F, Zhao C, et al. Treatment outcomes for different subgroups of nasopharyngeal carcinoma patients treated with intensity-modulated radiation therapy. *Chin J Cancer.* 2011; 30:565–573.

Appendix A List of abbreviations and definition of terms

Abbreviation or special term	Full form
NPC	nasopharyngeal carcinoma
EBV	Epstein-Barr virus
LA-NPC	locoregionally advanced nasopharyngeal carcinoma
CCRT	concurrent chemoradiotherapy
PF	cisplatin and fluorouracil
GTVp	primary gross tumor volume
SUV	standard uptake value
^{18}F-FDG-PET-CT	^{18}F-Fluorodeoxygenase positron emission tomography computed tomography
FFS	failure-free survival
OS	overall survival
DMFS	distant metastasis-free survival
LRRFS	locoregional relapse-free survival
KPS	Karnofsky performance status
AJCC/UICC	American Joint Committee on Cancer/International Union Against Cancer
ULN	upper limit normal
CT	computed tomography
MRI	magnetic resonance imaging
GTVn	gross tumor volume of involved lymph nodes
CTV1	high-risk clinical target volume
CTV2	low-risk clinical target volume
PTVs	planning target volumes
OARs	organs at risk
BSA	body surface area
CTCAE v4.0	Common Toxicity Criteria for Adverse Events version 4.0
RTOG	Radiation Therapy Oncology Group
FNA	fine needle aspiration
ICH	International Conference on Harmonization
GCP	Good Clinical Practice
SYSUCC	Sun Yat-Sen University Cancer Center
IRB	Independent Ethics Committee or Institutional Review Board
PS	Power and Sample Size Calculation
IQR	inter-quartile range
HRs	hazard ratios

Statistical Analysis Plan (version 1.0)

1 Overview

The study is a prospective, multicenter, open-label, randomized, controlled clinical trial.

The aim of this study is to demonstrate that adding adjuvant capecitabine to concurrent chemoradiotherapy (CCRT) could significantly improve the failure-free survival (FFS) in patients with locoregionally-advanced nasopharyngeal carcinoma (LA-NPC).

The null hypothesis is that adding adjuvant capecitabine to CCRT could not improve the FFS compared with CCRT-only in LA-NPC.

2 Sample size calculation

Sample size of this study is calculated using the Power and Sample Size Calculation (PS v3.1.2) software. A two-sided log-rank test with 54 overall events in both groups will provide at least 80% power to detect a hazard ratio (HR) of 0.46 when the 3-year FFS rate in the control group was 70% (estimated based on historical data) at a significance level of 5%. This HR would correspond to a 15.0% improvement in 3-year FFS with the addition of adjuvant capecitabine to CCRT. Considering a 4-year recruitment period and 3 years of follow-up, 164 patients (82 per group) will be needed to observe 54 FFS events, assuming that the distribution of survival times for both groups follow the exponential distribution. A total of 180 patients (90 per group) will be needed accounting for a drop-out rate of 10.0%.

3 Definitions of the outcomes

3.1 Primary outcome

The primary outcome is FFS, defined as time from date of randomization to documented relapse or death from any cause. Patients lost to follow-up or alive without disease relapse were censored at the date of last follow-up.

3.2 Secondary outcomes

Secondary outcomes will include the following:

1) Overall survival (OS), defined as time from date of randomization to death from any cause where patients lost to follow-up were censored at the date of last follow-up;
2) Distant metastasis-free survival (DMFS), defined as the time from date of randomization to documented distant metastasis or death from any cause;
3) Locoregional relapse-free survival (LRRFS), defined as the time from date of randomization to documented locoregional relapse or death from any cause;
   Patients with a distant metastasis as a first event will be censored for LRRFS at date of distant metastasis and vice versa; if both distant metastasis and locoregional relapse occurred at the same time, patients will be considered as having an event for both DMFS and LRRFS. Patients who are lost to follow-up or still alive without distant metastasis or locoregional relapse will be censored at the date of last follow-up;
4) Treatment compliance: the rate of patients who finished ≥2 cycles of concurrent cisplatin and ≥8 cycles of adjuvant capecitabine;
5) Treatment-related adverse events (TRAEs): TRAEs will be graded using the Common Toxicity Criteria for Adverse Events version 4.0 (CTCAE v4.0) for chemotherapy-related toxicities, and the Radiation Therapy Oncology Group (RTOG) radiation morbidity scoring criteria for radiotherapy-related toxicities. Delayed TRAEs post-treatment will be assessed annually using the RTOG radiation morbidity scoring criteria.

4 Statistical analysis

4.1 Analysis principles

1) For two-sided tests, the nominal level of type I error (α) will be 0.05 and the confidence level for two-sided confidence intervals (CI) will be 95%;
2) There will be no imputation of the missing values. The number of observations used in the analysis will be reported;
3) Intention-to-treat principle will be used to deal with the non-compliance;
4) Subgroup analyses will be carried out irrespective of whether there is a significant treatment effect on the primary outcome;
5) Analyses will be conducted primarily using SAS software SAS version 9.3 (SAS Institute Inc., Cary, NC) and R version 4.0.2 (http://www.r-project.org). Figures will be drawn using GraphPad Prism version 7.0.0 (GraphPad Software, San Diego, CA).

4.2 Data quality control

All responsible data collectors will be trained at the beginning of this study. An Electronic data capture (EDC) system will be used for data entry and management. The coordinator ensures that all data needed are collected.

4.3 Trial profile

The flow chart of inclusion and follow-up will be displayed in a diagram. The report will include the number of patients who met the inclusion criteria and the number included and reasons for exclusion of the non-included patients.

4.4 Patients characteristics

Description of the following baseline characteristics will be presented.

Discrete variables will be summarized by frequencies and percentages. Percentages will be calculated according to the number of patients for whom the data are available. The number of missing values will be added in a footnote in the corresponding summary table.

Continuous variables will be summarized by the use of standard measures of central tendency and dispersion, either mean and standard deviation [Mean ± SD], or median and 25%, 75% quartiles [Median(Q1-Q3)].

No statistical inference will be performed for the baseline variables.

Baseline measures for all patients will be tabulated.

4.5 Primary outcome

For the FFS, the data will be summarized by frequencies and percentages with 95% CI. The FFS will also be presented as Kaplan-Meier curves, compared using the log-rank test. Unstratified Cox proportional hazards regression models will be used to estimate hazard ratios (HRs) and corresponding 95% confidence intervals (CIs) will be calculated using the Wald test method. The proportional hazards assumption will be tested by including time-dependent covariates in the Cox models within the PROC PHREG module. Survival rates at 3- and 5-year will be reported with corresponding 95% CIs calculated using the log(-log) transformation of survival probabilities. Stratified Log-rank test and Cox regression will be also performed with different high-risk factors.

4.6 Secondary outcomes

For the OS, DMFS and LRRFS, the data will be summarized by frequencies and percentages with 95% CI, and will also be presented as Kaplan-Meier curves, compared using the log-rank test. Unstratified Cox proportional hazards regression models will be used to estimate hazard ratios (HRs) and corresponding 95% confidence intervals (CIs) will be based on the Wald test. The proportional hazards assumption will be tested by including time-dependent covariates in the Cox models within the PROC PHREG module. Survival rates at 3- and 5-year will be reported with corresponding 95% CIs calculated using the log(-log) transformation of survival probabilities.

For the rates of TRAEs will be presented in whole cohort.

4.7 Patients drop out

The intention-to-treat principle will be used to deal with the patients who dropped out.

4.8 Subgroup analysis

Subgroup analyses will be carried out for the primary and secondary outcomes.

Planned subgroup analysis:

- T3-4N2 or T1-4N3
- Plasma EBV DNA titer of >20,000 copies/mL
- Primary gross tumor volume (GTVp) of >30.0 cm^3
- ^{18}F-Fluorodeoxygenase positron emission tomography computed tomography (^{18}F-FDG-PET-CT) maximum standard uptake value (SUV_{max}) of GTVp of >10.0
- Multiple nodal metastases and any >4.0 cm
- Age category
- Gender: male, female
- Plasma EBV DNA titer post-CCRT

Forest plots will be constructed to illustrate subgroup analyses.

4.9 Tables and figures for the main paper

1) Table 1 will report the key baseline characteristics of the participants;
2) Table 2 will report the treatment summary in two groups;
3) Table 3 will report the incidence of AEs;
4) Figure 1 will show the flow chart of inclusion and follow-up;
5) Figure 2 will show the Kaplan-Meier plots of FFS, OS, DMFS and LRRFS;
6) Figure 3 will show a Forest plot of subgroups analyses.

A more extensive list of tables and figures used to report additional information is available upon request.
