# Supplementary material for: Adjuvant Capecitabine Following Concurrent Chemoradiotherapy in Locoregionally Advanced Nasopharyngeal Carcinoma: A Randomized Clinical Trial
Source: JAMA Oncol. 2022 Oct 13;8(12):1776–85. doi: 10.1001/jamaoncol.2022.4656 (PMC9562101; doi:10.1001/jamaoncol.2022.4656)
Supplement: Supplement 2. — eTable 1. List of Participating Centers eTable 2. Distribution of Patients With Different High-Risk Factors in Different Centers eTable 3. Dosimetric Outcomes of Radiotherapy Plans in Both Groups eTable 4. Compliance to Adjuvant Capecitabine eTable 5. Patterns of Failure eTable 6. Salvage Treatments After Relapse eTable 7. Incidence of Delayed Treatment-Related Adverse Events eFigure 1. Distribution of Patients With Different High-Risk Factors eFigure 2. Scaled Schoenfeld Residuals Plots for Different Survival End Points eFigure 3. Forest Plot for Failure-Free Survival for the Different Patient Subgroups eFigure 4. Failure-Free Survival for EBV DNA Titer End-CCRT [file jamaoncol-e224656-s002.pdf]

Supplementary Online Content

Miao J, Wang L, Tan SH, et al. Adjuvant capecitabine following concurrent chemoradiotherapy in locoregionally advanced nasopharyngeal carcinoma: a randomized clinical trial. Published online October 13, 2022. *JAMA Oncol*. doi:10.1001/jamaoncol.2022.4656

eTable 1. List of Participating Centers

eTable 2. Distribution of Patients With Different High-Risk Factors in Different Centers

eTable 3. Dosimetric Outcomes of Radiotherapy Plans in Both Groups

eTable 4. Compliance to Adjuvant Capecitabine

eTable 5. Patterns of Failure

eTable 6. Salvage Treatments After Relapse

eTable 7. Incidence of Delayed Treatment-Related Adverse Events

eFigure 1. Distribution of Patients With Different High-Risk Factors

eFigure 2. Scaled Schoenfeld Residuals Plots for Different Survival End Points

eFigure 3. Forest Plot for Failure-Free Survival for the Different Patient Subgroups

eFigure 4. Failure-Free Survival for EBV DNA Titer End-CCRT

This supplementary material has been provided by the authors to give readers additional information about their work.

© 2022 Miao J et al. *JAMA Oncology.*

Supplementary Tables

eTable 1. List of Participating Centers

Center	Principal investigator	Capecitabine group (n=90)	Control group (n=90)
SYSUCC	Chong Zhao	70 (50.4%)	69 (49.6%)
CHCAMS	Jun-lin Yi	12 (54.5%)	10 (45.5%)
JXCH	Jin-gao Li	8 (42.1%)	11 (57.9%)
NCCS[a]	Melvin L.K. Chua	-	-

Data are n (%).

[a] NCCS was involved in the trial design and steering committee.

Abbreviations: SYSUCC, Sun Yat-Sen University Cancer Center; CHCAMS, Cancer Hospital Chinese Academy of Medical Science; JXCH, Jiangxi Cancer Hospital; NCCS, National Cancer Centre Singapore.

© 2022 Miao J et al. *JAMA Oncology.*

eTable 2. Distribution of Patients With Different High-Risk Factors in Different Centers

Center		TNM stage classification[a]		SUV_{max} >10.0	Primary GTV >30.0 cm^3	Max LN diameter >4.0 cm	EBV DNA titer >20,000 copies/mL
		T3-4N2	T1-4N3				
**SYSUCC (n=139)**	Yes	61	29	106	103	11	25
	No	49		24	36	128	114
	Missing	-		9	-	-	-
**CHCAMS (n=22)**	Yes	10	2	18	22	1	3
	No	10		1	0	21	18
	Missing	-		3	-	-	1
**JXCH (n=19)**	Yes	6	8	9	14	4	3
	No	5		4	5	15	15
	Missing	-		6	-	-	1

[a] American Joint Committee on Cancer/Union for International Cancer Control 7th edition.

Abbreviations: SUV_{max}, maximum standard uptake value; GTV, gross tumor volume; Max LN, maximum lymph node; EBV, Epstein-Barr virus; SYSUCC, Sun Yat-Sen University Cancer Center; CHCAMS, Cancer Hospital Chinese Academy of Medical Science; JXCH, Jiangxi Cancer Hospital.

© 2022 Miao J et al. *JAMA Oncology.*

eTable 3. Dosimetric Outcomes of Radiotherapy Plans in Both Groups

	Capecitabine group (n=90)	Control group (n=90)
Dose of radiotherapy, Gy		
Primary planning target volume		
Prescribed dose	70 (70-70)	70 (70-70)
D98	70.5 (68.3-71.1)	70.3 (69.5-70.9)
D95	71.0 (69.9-72.7)	71.1 (70.2-71.4)
Lymph node planning target volume-left		
Prescribed dose	66 (64-68)	66 (64-68)
D98	65.9 (63.7-67.1)	66.2 (64.6-67.9)
D95	66.2 (64.4-67.7)	67.4 (65.3-68.5)
Lymph node planning target volume-right		
Prescribed dose	66 (64-68)	66 (64-68)
D98	66.6 (63.6-68.1)	67.2 (65.9-67.6)
D95	67.8 (64.5-68.9)	68.0 (67.0-68.2)
High-risk planning target volume		
Prescribed dose	60 (60-64)	60 (60-64)
D98	61.9 (61.4-63.5)	62.5 (60.9-64.0)
D95	63.8 (63.1-65.0)	64.8 (62.7-65.4)
Low-risk planning target volume		
Prescribed dose	54 (54-58)	54 (54-58)
D95	55.5 (54.8-58.3)	54.8 (54.2-59.2)
D98	55.0 (54.0-57.0)	53.9 (52.9-57.8)

Data are median (IQR).

© 2022 Miao J et al. *JAMA Oncology.*

eTable 4. Compliance to Adjuvant Capecitabine

	n=90
Patients receiving capecitabine	
0 cycle	5[a] (5.6%)
1 cycle	5 (5.6%)
2 cycles	3 (3.3%)
3 cycles	0
4 cycles	2 (2.2%)
5 cycles	2 (2.2%)
6 cycles	1 (1.1%)
7 cycles	1 (1.1%)
8 cycles	70 (77.8%)
13 cycles[b]	1 (1.1%)
Relative dose intensity, %	100.0 (84.7-100.0)
Patients with dose reduction	19 (21.1%)
Hand-foot syndrome	11 (12.2%)
Gastrointestinal AEs	4 (4.4%)
Hematological AEs	2 (2.2%)
Fatigue	1 (1.1%)
Non-compliance	1 (1.1%)
Patients with capecitabine discontinuation	14 (15.6%)
Disease progression	3 (3.3%)
Treatment-related AEs	4 (4.4%)
Drug allergy	1 (1.1%)
Non-compliance	6 (6.7%)

Data are n (%) or median (IQR).

[a] Five patients did not receive capecitabine, including 2 for AEs, 2 for patient withdrawal, and 1 having disease progression.

[b] One patient purchased his own adjuvant capecitabine off-protocol from another hospital.

Abbreviations: AEs, adverse events.

© 2022 Miao J et al. *JAMA Oncology.*

eTable 5. Patterns of Failure

	Capecitabine group (n=90)	Control group (n=90)
Local/regional relapse and distant metastasis	1 (1.1%)	3 (3.3%)
Local and/or regional relapse only	3 (4.4%)	11 (15.6%)
Local relapse	1 (1.1%)	2 (2.2%)
Regional relapse	2 (2.2%)	5 (5.6%)
Locoregional relapse	0	4 (4.4%)
Local and/or regional relapse patterns		
In-field	3	13
Unknown	1	1
Distant metastasis only	13 (14.4%)	15 (16.7%)
Distant metastatic sites		
Lung	7	10
Liver	1	7
Bones	8	6
Non-regional lymph nodes[a]	3	5
Others	1[b]	0
Unknown	1	0
Deaths	11 (12.2%)	17 (18.9%)
NPC-related	11 (12.2%)	13 (14.4%)
Non-NPC-related	0	2 (2.2%)
Unknown	0	2 (2.2%)

Data are n (%).

[a] Includes mediastinal, celiac or axillary lymph nodes metastasis.

[b] One patient had forehead subcutaneous and left zygomatic metastases.

Abbreviations: NPC, nasopharyngeal carcinoma.

© 2022 Miao J et al. *JAMA Oncology.*

eTable 6. Salvage Treatments After Relapse

	Capecitabine group (n=90)	Control group (n=90)
Treatments after locoregional relapse	4	14
Surgery +/- adjuvant chemotherapy or radiotherapy	2	2
Chemotherapy	1	3
Chemoradiotherapy	-	4
Anti-PD1 + chemotherapy or radiotherapy	1[a]	3
Traditional Chinese medicine	-	1
Unknown	-	1
Treatments after distant metastasis	14	18
Surgery	-	1
Chemoradiotherapy	1	3
Chemotherapy	3	8
Anti-PD1 +/- chemotherapy	7	2
Traditional Chinese medicine	-	2
Unknown	3	2

[a] One patient experienced locoregional relapse and distant metastases concurrently.

Abbreviations: anti-PD1, anti-programmed death 1 antibody.

© 2022 Miao J et al. *JAMA Oncology.*

eTable 7. Incidence of Delayed Treatment-Related Adverse Events

	Capecitabine group (n=83)[a]					Control group (n=81)[b]				
	Any grades	Grade 1	Grade 2	Grade 3	Grade 4	Any grades	Grade 1	Grade 2	Grade 3	Grade 4
Any AEs	79 (95.2%)	35 (42.2%)	35 (42.2%)	9 (10.8%)	0	76 (93.8%)	36 (44.4%)	33 (40.7%)	7 (8.6%)	0
Xerostomia	65 (78.3%)	49 (59.0%)	13 (15.7%)	3 (3.6%)	0	52 (64.2%)	40 (49.4%)	10 (12.3%)	2 (2.5%)	0
Subcutaneous fibrosis	60 (72.3%)	34 (41.0%)	23 (27.7%)	3 (3.6%)	0	53 (65.4%)	29 (35.8%)	23 (28.4%)	1 (1.2%)	0
Skin atrophy	59 (71.1%)	52 (62.7%)	7 (8.4%)	0	0	59 (72.8%)	49 (60.5%)	9 (11.1%)	1 (1.2%)	0
Hearing impairment	58 (69.9%)	32 (38.6%)	21 (25.3%)	5 (6.0%)	0	47 (58.0%)	26 (32.1%)	17 (21.0%)	4 (4.9%)	0
Temporal lobe necrosis	3 (3.6%)	3 (3.6%)	0	0	0	3 (3.7%)	2 (2.5%)	1 (1.2%)	0	0
Trismus	2 (2.4%)	1 (1.2%)	1 (1.2%)	0	0	3 (3.7%)	2 (2.5%)	1 (1.2%)	0	0
Cranial nerve injury	1 (1.2%)	1 (1.2%)	0	0	0	0	0	0	0	0
Brainstem injury	0	0	0	0	0	0	0	0	0	0
Thyroid malfunction	45 (54.2%)	45 (54.2%)	0	0	0	35 (43.2%)	34 (42.0%)	1 (1.2%)	0	0
Pituitary malfunction	52 (62.7%)	52 (62.7%)	0	0	0	50 (61.7%)	50 (61.7%)	0	0	0

Data are n (%).

[a] Seven patients had tumor relapse within 7 months post-CCRT.

[b] Eight patients had tumor relapse within 7 months post-CCRT, and one patient committed suicide after treatment.

The delayed treatment-related adverse events were assessed from 7 months post-CCRT until tumor relapse or last follow-up.

Abbreviations: AEs, adverse events.

© 2022 Miao J et al. *JAMA Oncology.*

Supplementary Figures

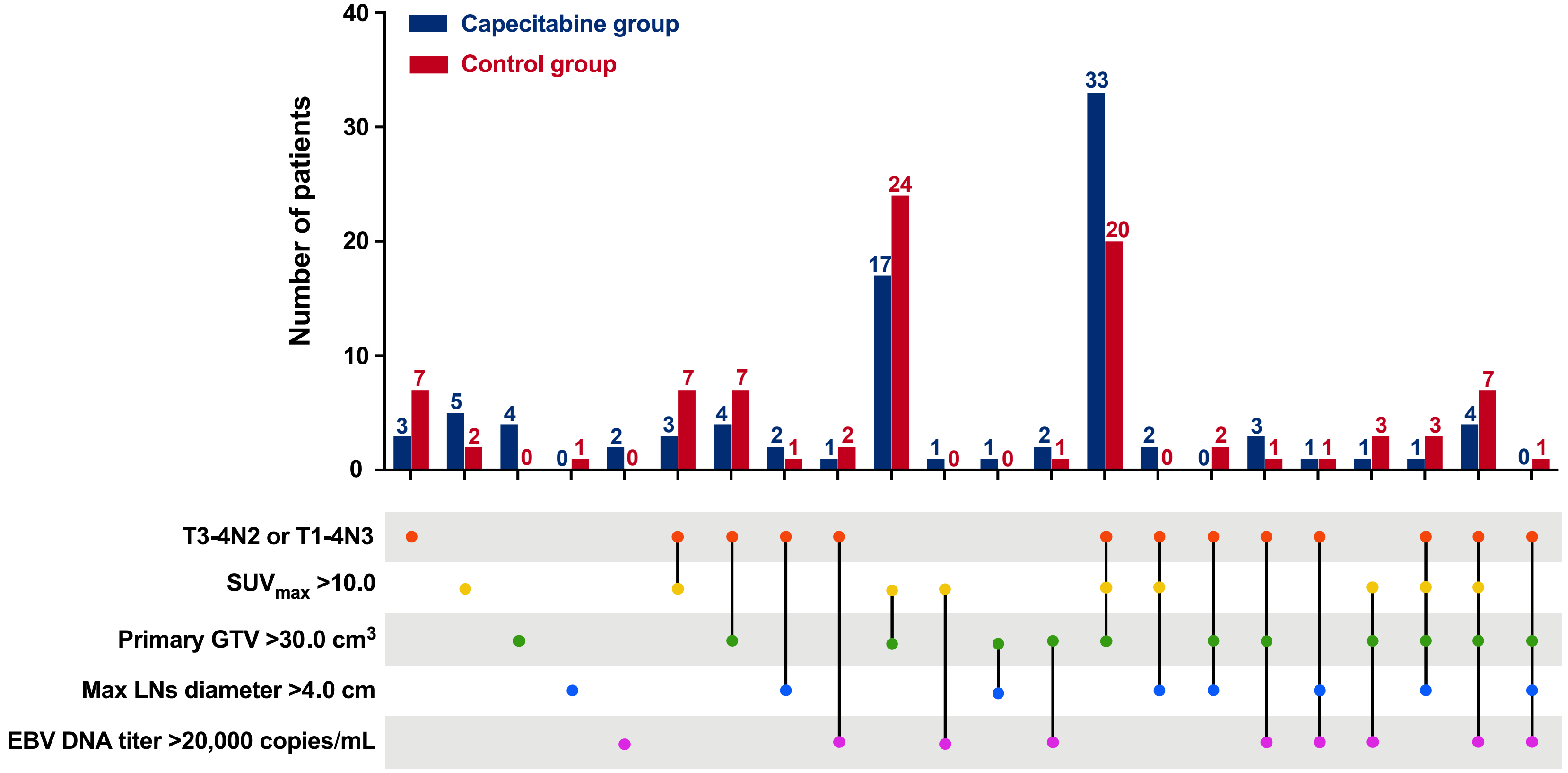

eFigure 1. Distribution of Patients With Different High-Risk Factors

Abbreviations: SUV_{max}, maximum standard uptake value; GTV, gross tumor volume; Max LN, maximum lymph node; EBV, Epstein-Barr virus.

© 2022 Miao J et al. *JAMA Oncology.*

(A) log-time time-scale

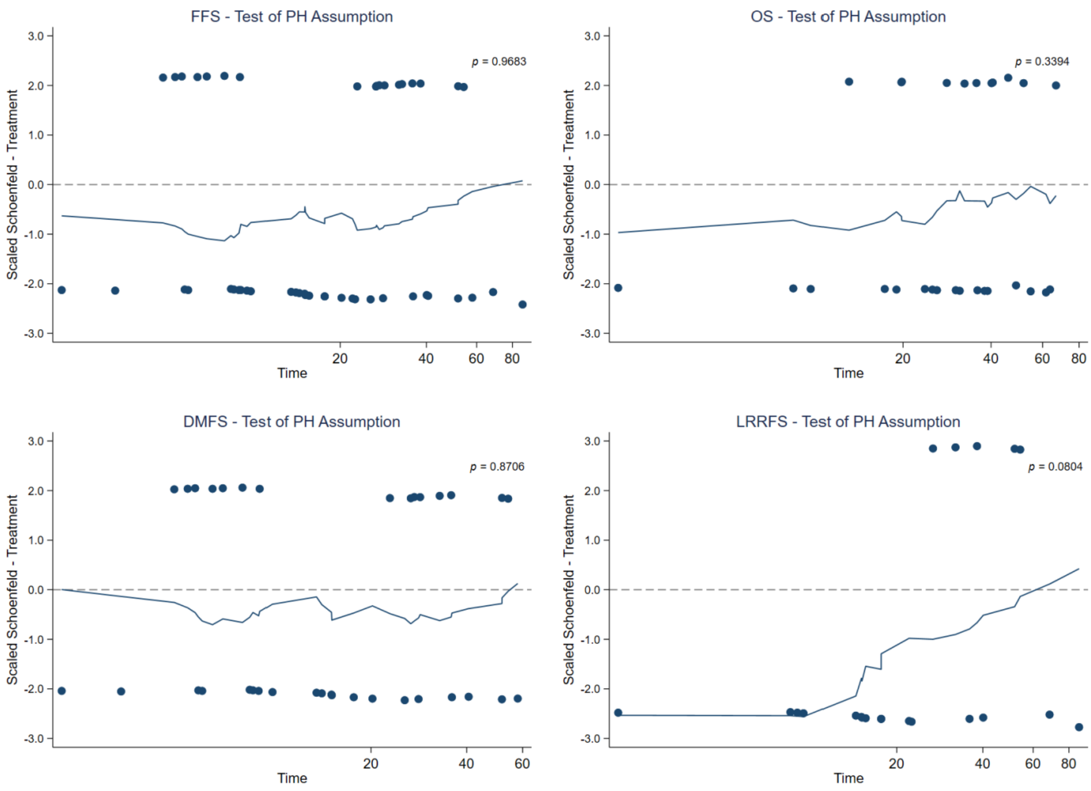

(B) linear time time-scale

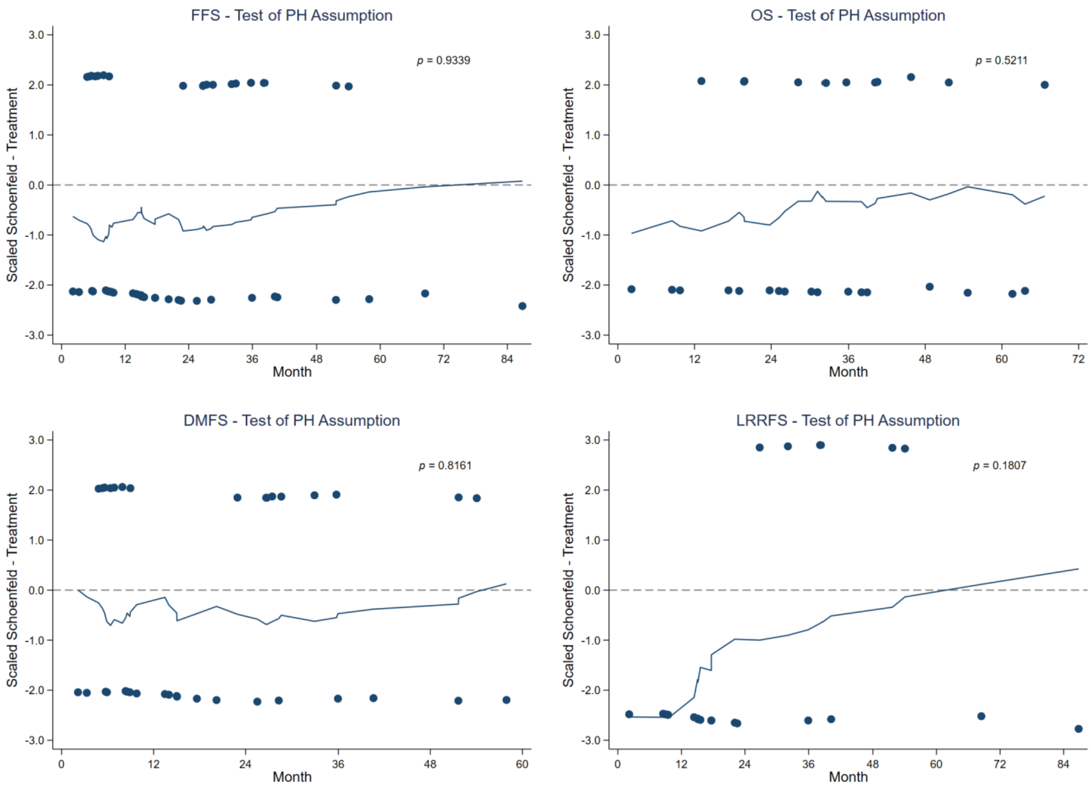

eFigure 2. Scaled Schoenfeld Residuals Plots for Different Survival End Points

Abbreviations: FFS, failure-free survival; OS, overall survival; DMFS, distant metastasis-free survival; LRFS, locoregional relapse-free survival; PH, proportional hazard.

© 2022 Miao J et al. *JAMA Oncology.*

Subgroup	No. of FFS events/total No. Capecitabine group	Control group	HR (95% CI)
**Overall**	18/90	31/90	0.53 (0.30-0.94)
Sex			
Female	3/17	4/20	0.81 (0.18-3.61)
Male	15/73	27/70	0.49 (0.26-0.91)
TNM stage			
All others	3/33	6/31	0.42 (0.10-1.67)
T3-4N2 or T1-4N3	15/57	25/59	0.60 (0.32-1.14)
SUV_{max}			
≤10.0	5/13	6/16	1.02 (0.31-3.34)
>10.0	10/67	19/66	0.46 (0.21-0.99)
Primary GTV			
≤30.0 cm^3	3/20	6/21	0.49 (0.12-1.95)
>30.0 cm^3	15/70	25/69	0.53 (0.28-1.01)
Max LN diameter			
≤4.0 cm	17/83	28/81	0.53 (0.29-0.98)
>4.0 cm	1/7	3/9	0.46 (0.05-4.41)
EBV DNA titer			
≤20,000 copies/mL	16/74	22/73	0.66 (0.35-1.26)
>20,000 copies/mL	2/15	9/16	0.20 (0.04-0.93)

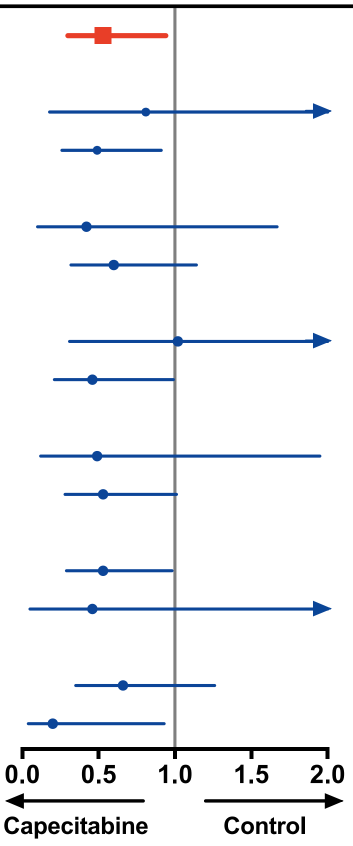

eFigure 3. Forest Plot for Failure-Free Survival for the Different Patient Subgroups

Abbreviations: FFS, failure-free survival; HR, hazard ratio; CI=confidence interval; SUV_{max}, maximum standard uptake value; GTV, gross tumor volume; Max LN, maximum lymph node; EBV, Epstein-Barr virus.

© 2022 Miao J et al. *JAMA Oncology.*

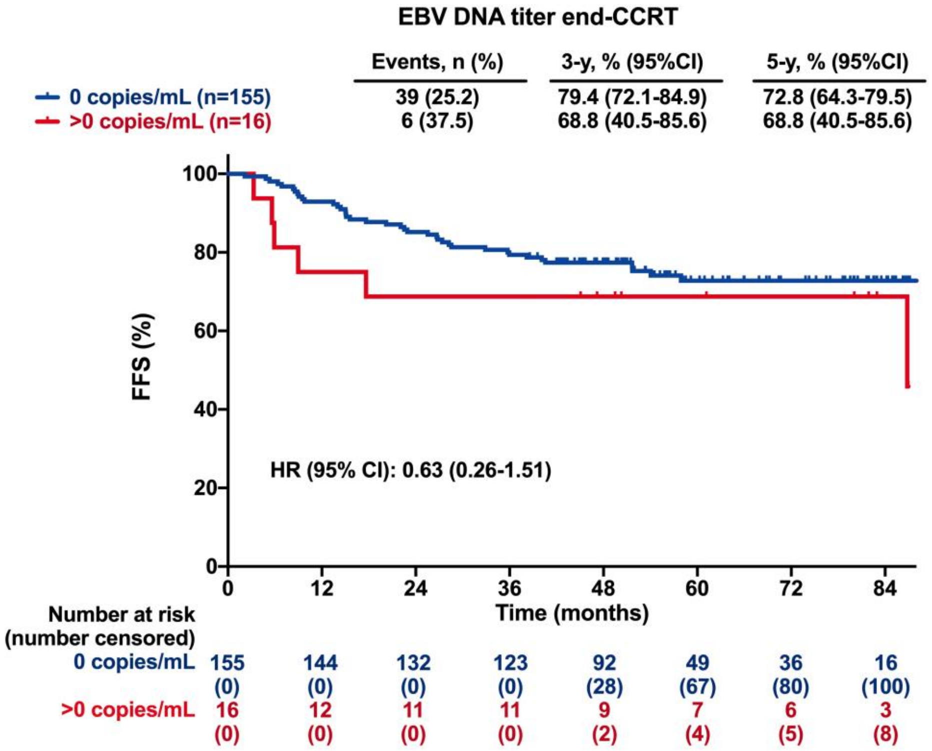

eFigure 4. Failure-Free Survival for EBV DNA Titer End-CCRT.

Abbreviations: FFS, failure-free survival; CCRT, concurrent chemoradiotherapy; HR, hazard ratio; CI, confidence interval; EBV, Epstein-Barr virus.

© 2022 Miao J et al. *JAMA Oncology.*
