# Supplementary material for: Adjuvant Capecitabine Following Concurrent Chemoradiotherapy in Locoregionally Advanced Nasopharyngeal Carcinoma: A Randomized Clinical Trial
Source: JAMA Oncol. 2022 Oct 13;8(12):1776–85. doi: 10.1001/jamaoncol.2022.4656 (PMC9562101; doi:10.1001/jamaoncol.2022.4656)
Supplement: Supplement 3. — Data Sharing Statement [file jamaoncol-e224656-s003.pdf]

Data Sharing Statement

Miao. Adjuvant Capecitabine Following Concurrent Chemoradiotherapy in Locoregionally Advanced Nasopharyngeal Carcinoma. *JAMA Oncol*. Published October 13, 2022. doi:10.1001/jamaoncol.2022.4656

Data
Data available: No

Additional Information
Explanation for why data not available: Research data can be requested from the corresponding author on reasonable request. Any request should be sent to the corresponding author, along with a detailed description of your research protocol. The corresponding author and Sun Yat-sen University Cancer Center will evaluate the reasonability of the request for our data and reserve the right to decide whether to share the data or not. Once approved, de-identified participant data will be made available.
